# Development of a Safe, Scalable, Course-Based Undergraduate Research Experience for Analytical Chemistry: The μCURE Project

**DOI:** 10.1021/acs.jchemed.5c00809

**Published:** 2025-08-15

**Authors:** Kimberley A. Frederick, Maury E. Howard, Kelly Y. Neiles, Daniel F. Scott, Rebecca A. Hunter

**Affiliations:** † Department of Chemistry, 7230 Skidmore College, Saratoga Springs, New York 12866, United States; ‡ Department of Chemistry and Biochemistry, 6044 Virginia Wesleyan University, Virginia Beach, Virginia 23455, United States; § Department of Chemistry, St. Mary’s College of Maryland, St. Mary’s City, Maryland 20686, United States; ∥ Department of Chemistry, 2779 Centre College, Danville, Kentucky 40422, United States; ⊥ Department of Chemistry, 3280 The College of New Jersey, Ewing, New Jersey 08628, United States

**Keywords:** Second-year undergraduate, Analytical chemistry, Laboratory instruction, Undergraduate research

## Abstract

Course-based undergraduate research experiences (CUREs) can have many positive effects on students’ learning and sense of self-efficacy. We have developed a networked CURE between four different institutions designed for courses in analytical chemistry that focuses on the process of adapting published solution-phase colorimetric assays into microfluidic paper analytical devices (μPAD) assays. We used a backward design process to develop 5 scaffolded learning outcomes: (1) identify and assess relevant literature sources, (2) propose a viable experimental plan to answer a well-defined scientific question based on literature information and experimental results, (3) apply appropriate methods of data analysis to interpret experimental results, (4) evaluate multiple pieces of experimental data to support conclusions, and (5) contribute to a team by working collaboratively toward common goals. Students begin the project by completing a literature search assignment to identify a published colorimetric assay they plan to adapt. They then write a proposal which identifies their analyte, sample of interest, and the figures of merit required for successful sample analysis using their μPAD. During the 3–5 weeks of laboratory work, students conduct their experiments, and each week evaluate the significance of their data and propose an experimental plan for the upcoming week. At the end of the μCURE project, students present their results in a joint, asynchronous poster session. Student artifacts are assessed for evidence of particular skills using rubrics from the Enhancing Learning by Improving Process Skills in STEM (ELIPSS) Project. Scores on the rubrics indicate partial to full attainment of each of the five learning outcomes.

## Graphical abstract

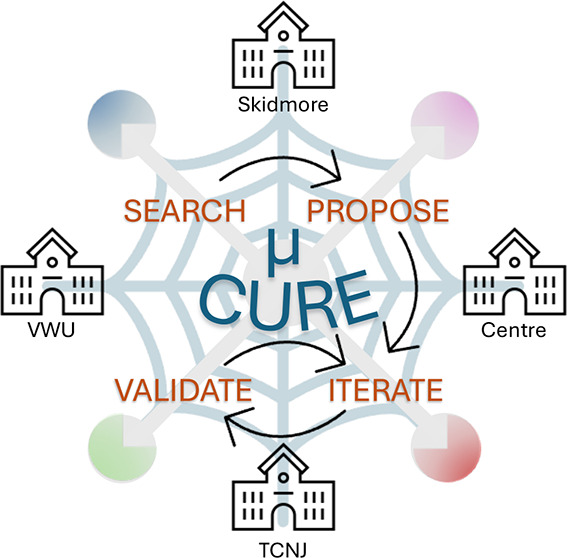

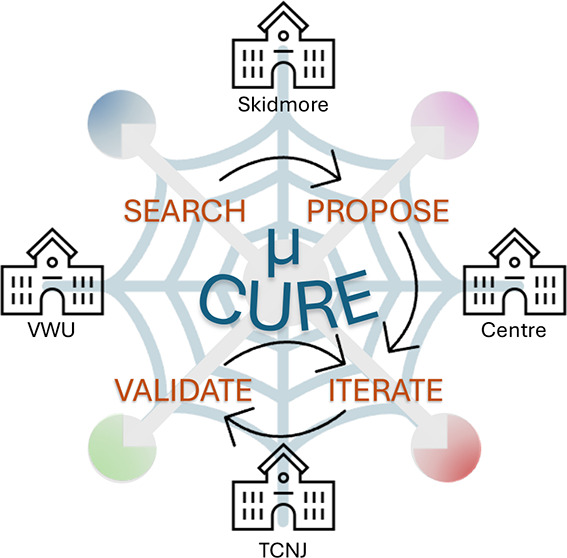

## Background

Over the last several decades, the benefits of undergraduate research for student’s persistence, scientific identity, and perception of their own learning have been well established, leading to calls for all students to have such experiences.^1,2^ In an effort to move away from the apprenticeship model and broaden research opportunities, there have been considerable efforts to develop course-based undergraduate research experiences (CUREs) across all levels of the curriculum.

Definitions of what constitutes a CURE and what is simply an inquiry-based lab can vary widely. For the purposes of this paper, we are adopting the definition of a CURE articulated by Dolan whereby: “whole classes of students address a research question or problem with unknown outcomes or solutions that are of interest to external stakeholders.” ^1,2^ Perhaps the most promising model for supporting implementation is that of a networked CURE involving multiple institutions. Networked CUREs overcome many of the most commonly cited barriers to implementation: time and resources. By dispersing the effort for CURE development, it is possible to both decrease the cost to any particular institution and to develop shared assignments and experimental protocols.^3–5^ For these reasons, we developed and implemented the μCURE project in a networked fashion across analytical chemistry courses at four different primarily undergraduate institutions.

The μCURE project focuses on the process of translating a traditional solution-phase assay into a paper microfluidic format. This is an active area of research because microfluidic paper-based analytical devices (μPADs) meet many of the dimensions of the World Health Organization’s REASSURED framework which specifies that diagnostic technology should provide real-time connectivity, ease of specimen collection, affordability, sensitivity, specificity, user-friendliness, rapid and robust results, equipment-free or simple operation, and deliverability.^6^ μPADs have recently proven to be a flexible and cost-effective platform for delivering inquiry-based laboratories in analytical chemistry.^7^ We have found this platform easy to learn and the technology has several key features which make it well suited to facilitate a CURE:

- It is inexpensive. This allows a student to use numerous devices, thus removing the fear of wasting a device on an experiment that might not work or not getting things perfect the first time. This also helps overcome cost as an often cited barrier to CURE implementation.^8^
- It produces results rapidly and without instrumentation. This allows a student to iterate; to get a result, evaluate its significance, and propose and complete a follow-up experiment within a single lab period.
- It is flexible, with three types of devices which are common: flow-through devices, surface tension enabled devices, and lateral flow devices as shown in Figure 1. All three of these devices can be produced on filter paper (Whatman 1 or Whatman 3).
- It produces data quickly and many parameters are reconfigurable. This allows students to evaluate the effects of different parameters on data and participate in the optimization process. Faculty can also provide different types of information and scaffolding to address their own learning outcomes.
- It is safe and produces minimal waste by using nano- to microgram quantities of reagents. This is important when students are given a greater degree of autonomy in experimental design.

**Figure 1:**
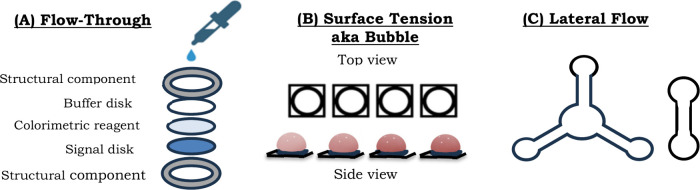
Illustration of the 3 different configurations of μPADs: (a) flow-through devices produced with a 0.5-in. hole punch, (b) surface tension enabled microfluidics (STEM) devices also known as bubble chips, and (c) lateral flow devices.

Because of these advantages, the number of published lab experiments that have employed paper microfluidics has grown dramatically in recent years. μPADs have been implemented in numerous courses from first year chemistry^9–14^ to upper-level courses.^15–17^ Most commonly μPADs have been used in analytical chemistry courses at different levels.^18–23^ The MICRO project developed an entire curriculum of μPAD-based laboratories to teach titrations, electrochemistry and colorimetry.^7,24^ Colorimetry, the approach used in this project, is the most common format for μPAD devices because of its relationship to commonly taught techniques like molecular absorbance and the availability of simple detector technologies. Experiments have been published that use scanners^17,25^ and cell phones as detectors.^18,26^ We have chosen to use cell phones because of their ubiquity and the flexibility to measure color intensity in a number of different color spaces (gray scale, RGB, CMYK, etc.).^20,26,27^

The overall scientific process is illustrated in Figure 2. In a traditional solution-phase assay, solutions are made in milliliter quantities and then absorbances are measured by a benchtop spectrophotometer to produce a calibration curve. In the μCURE project, reagents are deposited on filter paper (either hole punches or wax printed devices) and then dried. Standard and/or sample solutions are then added onto the paper where the color producing reaction happens. The intensity of the resulting color is proportional to the amount of product formed if the target analyte is the limiting reagent. The relationship between the measured color and the analyte concentration is determined using a calibration curve produced from a set of standards. Images are captured using a smartphone and then analyzed using free digital imaging software to measure their RGB values which are then used to produce calibration curves. Using this data students determine the figures of merit required to properly analyze their sample of interest for their particular analyte (e.g., levels of arsenic in groundwater). These figures of merit include sensitivity, detection limit, linear range, accuracy, precision, and selectivity. At the end of the project, students compare the figures of merit they measured with those needed for their sample analysis to determine if their goals were achieved.

**Figure 2:**
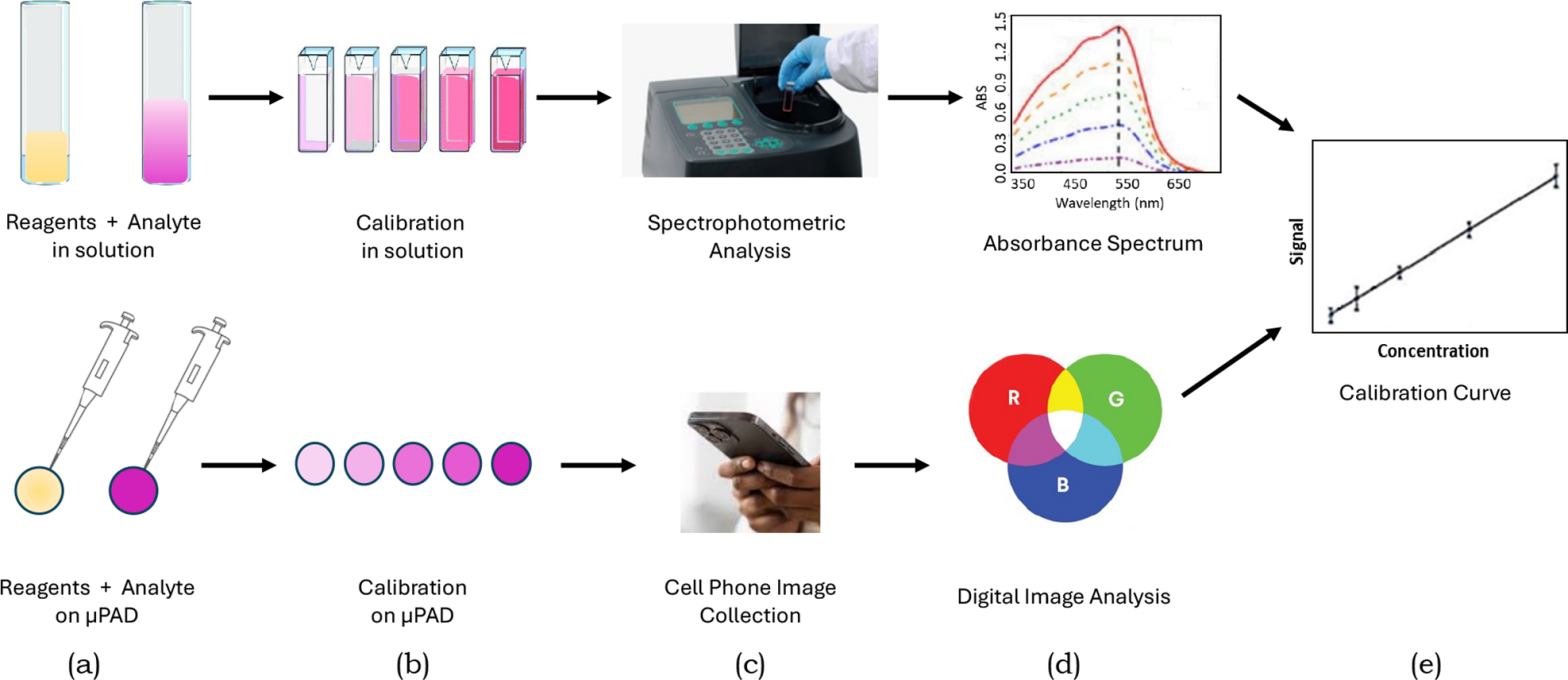
Comparison of solution phase versus μPAD assay: (a) standards and samples are mixed with color producing reagents; (b) standards produce responses with a gradient of color based on analyte concentration; (c) color is measured either with spectrophotometer or cell phone image and then (d) analyzed for absorbance or color value, and (e) both produce calibration curves.

## Overview of μCURE Learning Goals and Structure

To develop the μCURE, the four analytical authors were guided by the chemical education coauthor through the use of backward design to generate a set of aligned student learning objectives for the project.^28,29^ Our goal was for these objectives to be specific to the CURE while also being more broadly transferrable to future research experiences as well as science courses more generally. The shared outcomes are as follows:

- Identify and assess relevant literature sources.
- Propose a viable experimental plan to answer a well-defined scientific question based on literature information and experimental results.
- Apply appropriate methods of data analysis to interpret experimental results.
- Evaluate multiple pieces of experimental data to support conclusions.
- Contribute to a team by working collaboratively toward common goals.

Each outcome maps onto a specific element of the μCURE, as well as one or more specific process skills/science practices, as summarized in Figure 3.

**Figure 3:**
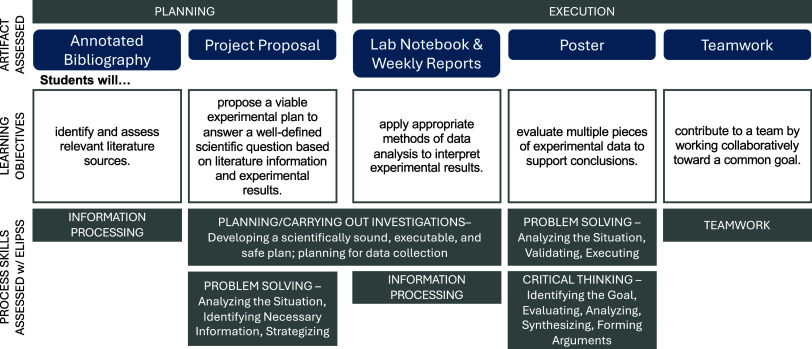
Major components of the μCURE, along with their aligned learning objectives and corresponding process skill assessment rubric. Planning activities took place in parallel with traditional experiments and primarily outside of scheduled laboratory time. The execution activities took place during 3–5 weeks of scheduled lab time. Student artifact is most often an assignment that is turned in and assessed with a rubric.

The analytical coauthors took these learning outcomes and built lab activities for the semester such that the μCURE projects were the culminating experience. The μCURE projects were implemented at each institution in the 2023–24 academic year in a pilot format. Following pilot implementation, the coauthors reflected on their experiences and various forms of student learning data to inform adjustments to the experiences. Updated μCURE experiences were then implemented during the 2024–25 academic year (Table 1). The materials presented here, including student learning data, are from these second iterations of updated experiences.

**Table 1:** Details of the μCURE Implementation at the Four Different Institutions

Institution	Course Identifier	Semester of Updated CURE Implementation	*n*	Student Year	Student Majors	CURE Length^a^
Skidmore	CH 232	Spring 2025	10	2nd, 3rd, and 4th	Chemistry, biochemistry	5 weeks
TCNJ	CHE 310	Spring 2025	19	2nd, 3rd, and 4th	Chemistry, premed (bio, psych, etc.)	5 weeks
VWU	CHEM 210	Fall 2024	8	2nd, 3rd, and 4th	Chemistry, biochemistry (some biology, EES)	4 weeks
Centre	CHE 250	Fall 2024	19	2nd, 3rd, and 4th	Chemistry, biochemistry and molecular biology	5 weeks

a Refers to number of weeks of lab work and does not include other aspects of the CURE that may be due before lab work begins (e.g., the proposal).

## Integrating Into the Curriculum and Assessment of μCURE Elements

Implementation of the μCURE followed a consistent pedagogical structure across all participating institutions, integrating traditional analytical laboratory instruction with the scaffolded research experience. While students were introduced to the premise of the μCURE as early as the first class meeting, laboratory work directly related to the project was concentrated in the final third of the semester. During the earlier weeks of the semester, students engaged in foundational analytical chemistry experiments that reinforced core concepts such as accuracy and precision, pipetting, solution preparation, calibration, titration, and chromatography. These exercises also aimed to develop essential process skills/scientific practices, including experimental planning and critical thinking. One of these early laboratories also introduced students to analytical method development through a paper-based iron assay,^18^ providing an initial experience with paper microfluidics.

Planning activities for the μCURE—including a review and proposal development—helped students identify an analyze/sample of interest and design their experiments around relevant figures of merit. These planning assignments ran in parallel with traditional laboratories and were conducted outside of the scheduled lab period. Each μCURE had a common goal: translating a solution-phase spectrophotometric assay into a paper microfluidic format using smartphone-based detection. Students employed standard device formats—typically flow-through or surface-tension mediated designs—and evaluated their assays using consistent performance metrics such as linear range, sensitivity, stability, and selectivity. Depending on institutional schedules, a virtual poster presentation occurred during the final week of the semester or exam period, allowing students to present their research to a broader academic audience. All assignments and their associated rubrics and supporting materials are provided in the Supporting Information (SI). Key assignments are also summarized in Figure 2. The implementation timeline for each institution is summarized in Table 2 below.

**Table 2:**
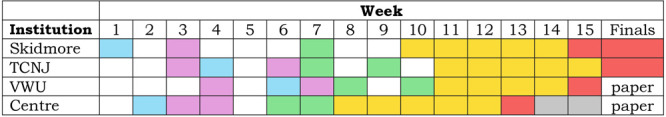
Timeline for Implementation of CURE-Related Assignments^a^

a Team contract (blue), literature search (purple), proposal (green), weekly progress reports/notebooks (yellow), and final poster (red). For some institutions, the literature search and proposal may appear more than once in the timeline, indicating students submitted a draft and received feedback prior to a final submission.

As teamwork was a central learning outcome, each group completed a team contract early in the process (prior to drafting the proposal) to establish expectations and guide their collaboration. This document also served as a reference in the event of group conflict. Teamwork was assessed during the experimental phase using the ELIPSS teamwork rubric.^30,31^

In order to identify the sample to study and the associated solution-phase assay to adapt, students conducted a literature search related to the analyte and/or sample of their choosing, and summarized key points from the most relevant sources in the format of an annotated bibliography. The assignment included keyword guidance (e.g., “colorimetric” recommended; “nanoparticle,” “enzyme” discouraged). Based on how the literature search fits into the curriculum at each institution, this assignment may include more or less emphasis on learning how to search the literature, how to read and evaluate articles, and/or how to summarize the most important points. Student products were assessed using the ELIPSS information processing rubric.^30^ Both the assignments and the rubric can be found in the .

Following the bibliography, students developed their research proposal. Structured prompts within the assignment, provided in the , guided students through the experimental design process. For example, to get the students thinking about what standard concentrations they should create and test prior to beginning their experiments, they were asked what concentration range of analyte would be expected in their chosen sample, and were required to provide a literature reference to support their answer. At some institutions, the proposal was first submitted as a draft and revised in response to instructor feedback with a “response to reviewer.” Students also constructed a flowchart to visualize their experimental plan. Proposals were evaluated using elements of the ELIPSS planning and carrying out investigations rubric and elements of the problem-solving rubric (analyzing the situation, strategizing, identifying necessary information).^30^

Once laboratory work began, students submitted weekly progress reports reflecting on experimental outcomes, identifying which aspects of their plan succeeded or failed, and proposing adjustments. These reports served as prelab assignments for the following week and helped students iteratively refine their procedures. These weekly updates were evaluated using the same planning and carrying out investigations rubric used to evaluate the proposal as well as the ELIPSS information processing rubric.^30^

At the culmination of the project, students prepared a scientific poster detailing both their methods and results. A networked CURE provides a unique opportunity for students to share their work with peers at other institutions, as well as learn more about the work of others. The virtual nature of the poster session enabled asynchronous presentation and feedback, using Google Slides with embedded audio and comments. The instructions for preparing the poster can be found in the . This final artifact of the μCURE was evaluated using multiple elements of ELIPSS rubrics: problem solving (analyzing, validating, executing) and critical thinking (identifying goals, evaluating, synthesizing, and forming arguments).^30^

## Sample μPAD Results Based on Figures of Merit

While the nature of research is most certainly nonlinear and driven by the particular μPAD that is being developed, there are certain experiments and approaches that are common across institutions and student groups. In our description of this process, we will use one example project, the determination of acetylsalicylic acid (ASA) in commercial aspirin tablets through complex formation with ferric ions.

The students typically began by determining the type of μPAD configuration they would like to use. In the μCURE project, we generally used flow-through and surface tension enabled devices to limit the number of experimental parameters that needed to be optimized (e.g., channel dimensions of lateral flow devices). Flow-through devices are the easiest to produce and use as they are made with a 0.5-in. hole punch and the fluid is confined on the chip by the bounds of the paper. Both surface tension enabled device and lateral flow devices contain and direct flow using hydrophobic barriers. Lateral flow devices are interesting to students because of their experiences with common tests using lateral flow such as COVID or pregnancy tests. But, they can also be trickier to use because the reagents need to be immobilized so that the sample solution can flow past it. In the aspirin μPAD example, students chose to use the surface tension enabled configuration because they are easier to use than the lateral flow pads but can give better results than the hole punch devices. Confining the area of reaction to a definite cross-section is analytically advantageous.

Students began by running the assay on the microfluidic device or in a microcentrifuge tube using the published protocol they were adapting. Because the microfluidic devices typically hold a maximum of 150 μL, it was necessary to scale down the volumes and/or increase the concentrations from the reagents in the original publication. Once initial results were obtained, it was possible to make a decision on what color channel to use. The most common four options are red, green, blue or black (which is done using gray scale analysis). The results in Figure 4 show the color channel data for the aspirin project. The data is evaluated based on maximizing the slope of the calibration curve which will ultimately be related to sensitivity. It is typical that at least one of the color channels produces little to no response and can quickly be eliminated. Calibrations curve slopes are generally negative because of the way that cell phones record color with white having the highest values and darker colors have smaller color values. Based on the data in Figure 4, the students chose to use the red channel. While it may be possible to predict which color channel to use based on the color change, it is often better practice to determine the channel using experimental data.

**Figure 4:**
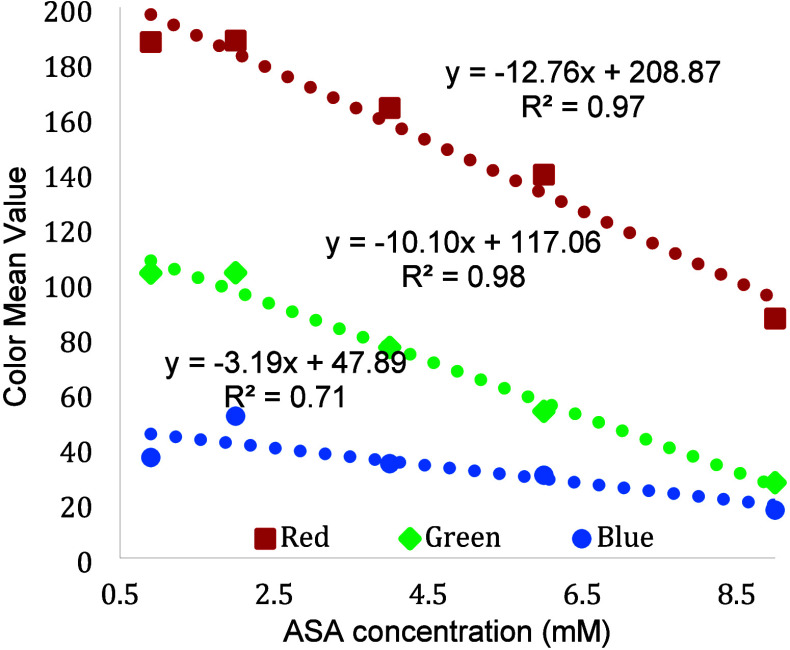
Data used to determine optimal color channel from cell phone images analyzed using ImageJ software. Based on slope and *R* ^2^ value, red was determined to be the best color channel.

The next experiment usually involves determining the optimum time from depositing the sample on the chip to making the color measurement. This is important because it can take time for chemicals to migrate within the paper matrix and react. On the other hand, once the colored complex forms it can also break down. This means that it is important to know both how long to wait and if there is a window when the color response is stable. An example of the time stability data can be found in Figure 5. As can be seen from the data, the iron salicylate complex forms quickly and is quite stable, so time is not an important variable for this particular assay. The data from this experiment is a good learning opportunity to discuss how much variation with time is important in regards to the response of the assay (is a change in color value of 2 important? Or a change of 5?) and what the appropriate time frame is for this experiment (i.e., how long do you expect it to take to deposit your sample, mix it and then measure the color value?).

**Figure 5:**
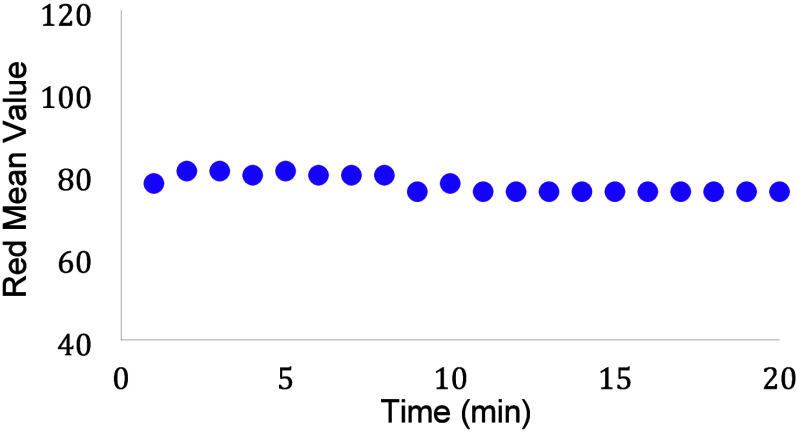
Measurements of color value every minute to determine the best reaction time. In this case, the reaction happens during the first minute and remains consistent for up to 20 min.

Because the students have proposed μPADs for testing a specific analyte in a specific matrix, much of the remaining experimental work is focused on the determination and optimization of the figures of merit that must be met in order to properly analyze the particular analyte in the chosen sample of interest. These figures of merit include sensitivity, limit of detection/quantitation (LOD/LOQ), linear range, linearity, accuracy, precision, and selectivity/specificity. Before beginning laboratory work, students can make general statements about appropriate requirements for each figure of merit for a given application based on the literature (e.g., the LOD/LOQ should fall below the expected analyte concentration in the sample of interest, a linear range that encompasses the expected range of analyte concentration is ideal). As depicted in Figure 6, students typically work through an iterative cycle in the lab where they construct a calibration curve, run some samples, and analyze the data. The figures of merit from this first pass typically do not meet required specifications, so students must decide what to modify in an attempt to improve their results. For example, if the LOD is too high, students might try to enhance the sensitivity by optimizing the chemistry (e.g., increasing reagent concentration, increasing sample volume, considering pH effects) or changing another aspect of the device (e.g., smaller devices might result in a more obvious color change). At the end of the project, students compare the figures of merit they measured with those needed for their sample to determine if their goals were achieved.

**Figure 6:**
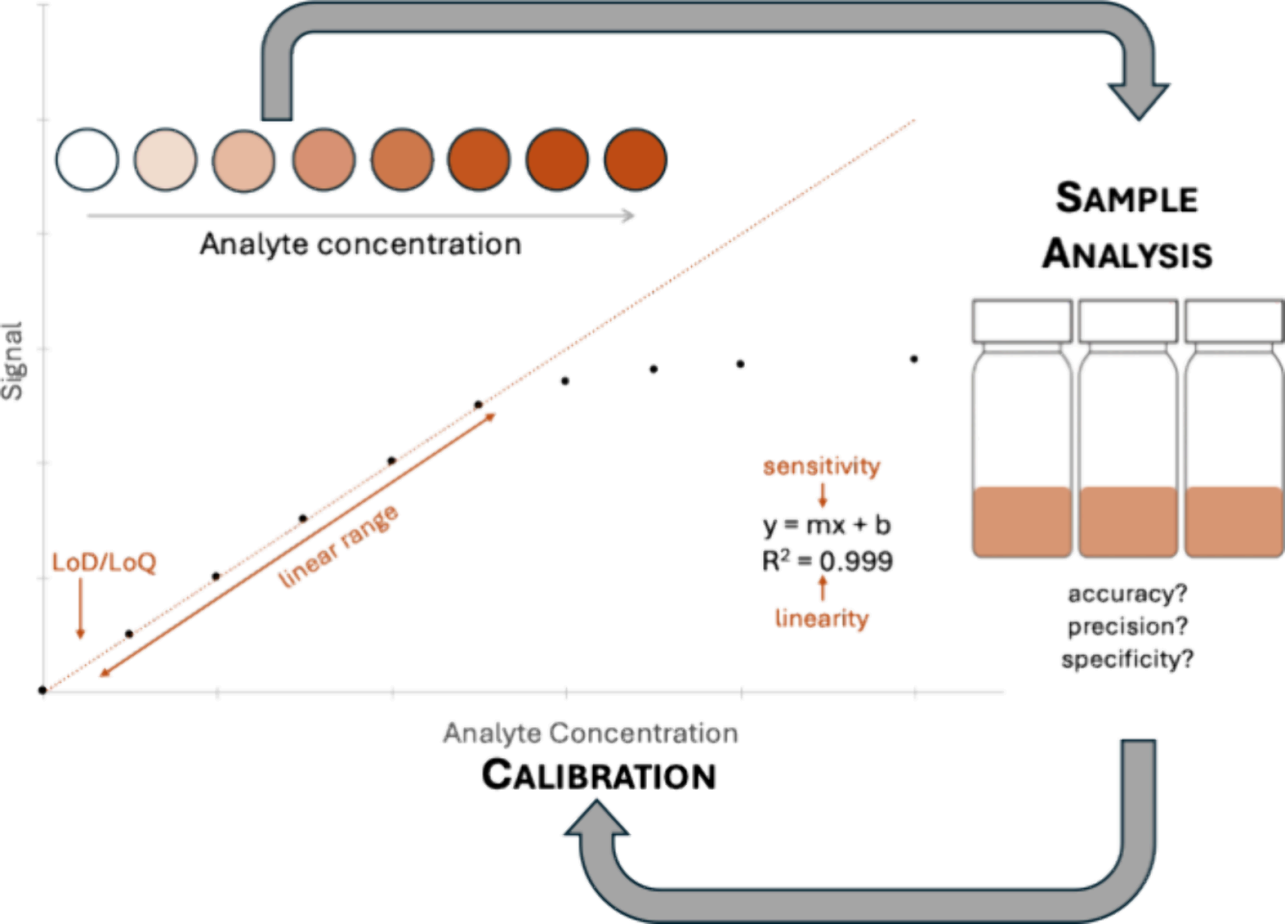
Summary of the iterative cycle of laboratory work for characterization of μPAD performance.

In the aspirin example, students optimized the sample pretreatment strategies including boiling time. They also optimized the concentrations of ferric and counterions (chloride and nitrate). Students also tested the impact of their sample coating color and binding materials on the assay results to assess the specificity of the assay. A summary of the figures of merit measured can be found in Table 3 below.

**Table 3:** Figures of Merit Determined for the Aspirin Assay

Figure of Merit	Value
Linear Range	8–24 mM
Sensitivity	6.3 mM^–1^
Accuracy (% error)	3%
Precision (%RSD)	1%
Specificity	No interferences observed

## Student Learning Data

All student learning data was collected in accordance with protocol approved by the IRB of each implementing institution. During the second implementation (academic year 2024–25) all five learning outcomes were assessed using ELIPSS rubrics.^30,31^ The name of the ELIPSS rubric used for each learning outcome, what artifact was assessed, and the number of students assessed is summarized above in Figure 2. Each table below reports the results for a single learning outcome. In all cases, the scores represented evidence shown for the category within the artifact assessed and ranged from 1 (minimally/inaccurately) to 5 (completely/accurately) with 0 representing no evidence found.

The data in Table 4 show that students at all four institutions are performing, on average, above a 3 on all four categories of the information processing rubric when it was applied to the literature search assignment. This indicates that the artifacts contained evidence of partial to complete student ability to identify and assess relevant literature sources.

**Table 4:** Learning Data for Outcome 1: Identify and Assess Relevant Literature Sources

Rubric Category^a^	TCNJ Avg (S.D.)	Skidmore Avg (S.D.)	VWU Avg (S.D.)	Centre Avg (S.D.)
Number of Groups (*n*)	6	4	3	7
Evaluating	3.8	3.8	3.8	4.7
	(1.6)	(0.9)	(0.8)	(0.5)
Interpreting	3.7	4.9	4.0	4.1
	(1.2)	(0.3)	(0.00)	(0.7)
Manipulating/Transforming (Extent)	4.3	4.4	5.0	3.9
	(0.8)	(0.6)	(0.00)	(1.1)
Manipulating/Transforming (Accuracy)	4.3	4.4	3.7	3.9
	(0.8)	(0.8)	(0.6)	(1.1)

a Artifact assessed: Literature search assignment. ELIPSS rubric used: Information processing feedback.

The data in Table 5 below show that in the majority of the cases, students at all four institutions are performing, on average, above a 3 on the three categories used to assess their ability to create viable experimental designs. This indicates that the artifacts contained evidence of partial to complete student understanding of these skills. There were, however, five cases where the scores dropped below a 3, indicating an incomplete or incorrect understanding of the skill.

**Table 5:** Learning Data for Outcome 2: Propose a Viable Experimental Plan to Answer a Well-Defined Scientific Question Based on Literature Information and Experimental Results

Rubric Category^a^	TCNJ Avg (S.D.)	Skidmore Avg (S.D.)	VWU Avg (S.D.)	Centre Avg (S.D.)
Number of Groups (*n*)	6	5	3	7
Analyzing the Situation (PS)	4.0	3.8	2.7	4.0
	(1.1)	(0.8)	(0.6)	(1.0)
Identifying (PS)	3.0	3.6	3.0	3.4
	(0.0)	(0.5)	(0.9)	(1.0)
Strategizing (PS)	3.3	4.0	2.5	3.3
	(1.5)	(0.7)	(0.5)	(1.4)
Sound Plan (PCOI)	2.7	4.2	3.3	4.1
	(1.5)	(0.8)	(0.6)	(0.9)
Data Collection (PCOI)	3.3	4.0	3.3	3.4
	(1.5)	(1.0)	(0.6)	(1.1)
Executable (PCOI)	2.3	3.8	4.0	3.9
	(1.6)	(0.8)	(0.0)	(1.2)
Safe (PCOI)	4.3	N/A	2.7	4.4
	(1.0)		(0.6)	(0.8)

a Artifact assessed: Project proposal. ELIPSS rubric used: Problem solving (PS) feedback and planning/carrying out investigations (PCOI) feedback.

The data in Table 6 below show that in the majority of the cases, students at three of the four institutions are performing, on average, above a 3 on the four categories used to assess their ability to process information. This indicates that the artifacts contained evidence of partial to complete student understanding of these skills. At the fourth institution, VWU, the averages for each category was between 2.5 and 3.1, indicating only partial understanding of these skills.

**Table 6:** Learning Data for Outcome 3: Apply Appropriate Methods of Data Analysis to Interpret Experimental Results

Rubric Category^a^	TCNJ Avg (S.D.)	Skidmore Avg (S.D.)	VWU Avg (S.D.)	Centre Avg (S.D.)
Number of Groups (n)	6	5	3	7
Evaluating (IP)	3.1	3.7	2.7	3.3
	(0.6)	(0.9)	(1.0)	(0.9)
Interpreting (IP)	2.8	4.2	2.3	3.1
	(0.8)	(0.8)	(0.5)	(1.3)
Manipulating/Transforming—Extent (IP)	3.6	3.9	2.6	3.1
	(1.1)	(0.8)	(0.9)	(1.2)
Manipulating/Transforming—Accuracy (IP)	3.8	4.5	2.8	2.8
	(0.8)	(0.8)	(1.5)	(1.2)
Sound Plan (PCOI)	3.1	3.9	3.1	3.2
	(1.4)	(0.7)	(0.3)	(1.3)
Data Collection (PCOI)	3.0	3.5	2.9	3.4
	(1.6)	(1.5)	(0.8)	(1.2)
Executable (PCOI)	2.9	3.8	2.5	3.0
	(1.2)	(0.9)	(0.9)	(1.3)

a Artifacts assessed: Lab notebook and weekly reports. ELIPSS rubric used: Information Processing (IP) Feedback and Planning/Carrying Out Investigations (PCOI) Feedback

The data in Table 7 below show that with only a few exceptions, students at all four institutions are performing, on average, above a 3 on all categories of the problem solving and critical thinking rubric when they were applied to the poster assignment. This indicates that the artifacts contained evidence of partial to complete student understanding of these skills.

**Table 7:** Learning Data for Outcome 4: Evaluate Multiple Pieces of Experimental Data to Support Conclusions

Rubric Category^a^	TCNJ Avg (S.D.)	Skidmore Avg (S.D.)	VWU Avg (S.D.)	Centre Avg (S.D.)
Number of Groups (*n*)	6	5	3	7
Analyze the Situation (PS)	3.9	4.8	3.3	3.4
	(0.7)	(0.4)	(0.6)	(1.1)
Validating (PS)	3.8	3.2	3.7	3.0
	(0.8)	(1.4)	(0.6)	(0.8)
Executing (PS)	3.5	4.2	3.3	2.9
	(0.8)	(0.9)	(0.6)	(1.5)
Identify the Aim (CT)	3.9	4.8	3.6	4.0
	(0.7)	(0.4)	(0.6)	(0.8)
Evaluating (CT)	4.0	4.0	3.3	2.6
	(1.2)	(0.6)	(0.6)	(0.8)
Analyzing (CT)	3.5	4.1	3.0	3.6
	(0.8)	(0.7)	(1.0)	(1.3)
Synthesizing (CT)	4.3	3.9	2.3	3.0
	(0.5)	(0.7)	(0.6)	(0.8)
Argument (Structure) (CT)	2.7	3.7	2.3	3.1
	(1.6)	(0.7)	(0.6)	(1.2)
Argument (Validity) (CT)	4.1	4.5	2.8	3.3
	(1.1)	(0.5)	(0.8)	(1.4)

a Artifact assessed: Poster. ELIP SS rubric used: Problem Solving (PS) and Critical Thinking (CT) Feedback

The data in Table 8 below show that students at all four institutions are performing, on average, above a 3 on all four categories of the teamwork rubric when it was applied to the teamwork aspects of their assignments. This indicates that the artifacts contained evidence of partial to complete student understanding of these skills.

**Table 8:** Learning Data for Outcome 5: Contribute to a Team by Working Collaboratively toward Common Goals

Rubric Category^a^	TCNJ Avg (S.D.)	Skidmore Avg (S.D.)	VWU Avg (S.D.)	Centre Avg (S.D.)
Number of Groups (*n*)	6	5	3	7
Interacting	3.9	4.3	3.7	3.3
	(0.7)	(0.8)	(0.6)	(1.1)
Contributing	3.7	3.7	4.0	3.4
	(0.6)	(1.0)	(0.4)	(1.1)
Progressing	3.8	3.4	3.8	3.1
	(0.9)	(1.0)	(0.2)	(1.2)
Building Community	4.0	4.4	3.5	3.7
	(1.0)	(0.4)	(0.3)	(1.0)

a Artifact assessed: Teamwork. ELIPSS rubric used: Teamwork feedback.

Overall, the results of the analysis on student learning data indicate that student learning artifacts are indeed showing evidence of the scientific practices targeted by the CURE experiences. While some areas may need additional attention in future implementations, the CUREs seem to be having the intended student learning outcomes.

## Discussion of Lessons Learned during Implementation

As noted, participation in the μCURE project clearly provided deep learning opportunities for students. While many of the , assignments, laboratories, and resources can be found embedded in the materials in the , a few key tips are highlighted below.

Instructor familiarization with the possible fabrication techniques will aid in the planning and facilitating of the μCURE. μPADs can be fabricated from a range of methods, including wax printing, inkjet printing, photolithography, or cutting/stenciling. Each fabrication method provides advantages and disadvantages in terms of ease of use, performance, adaptability, cost, accessibility, etc. but are all also capable of producing μPADs.

There are also several types of solution-based assays that decrease the likelihood of successful translation to a paper-based format. Assays that involve organic solvents are not successfully contained using hydrophobic barriers and should therefore be avoided. Assays requiring extensive sample pretreatment (e.g., liquid–liquid extractions, solvent evaporation) are also not ideal because they potentially undermine the low cost and ease of use for ideal μPADs. Assays that use nanoparticles or colorimetric reagents that cannot be purchased commercially may be beyond the scope of an analytical chemistry course, requiring additional time for preparation and troubleshooting.

While the start of the lab time devoted to the μCURE can be tailored to instructor and institution preferences, scaffolding and extending the preparation, skills, and learning objectives beyond the “hands on” lab weeks has been beneficial. Allowing students to complete additional assignments and lab experiments focusing on smaller parts of the μCURE helps the students prepare for the project. Scaffolding lab skills has proven valuable, with laboratories exposing students to standard solution and reagent preparation, calibration curves, μPADs, ImageJ utilization, and the translation of a solution-based protocol to paper-based prepare students well for the μCURE project.

The timing of μCURE-specific supporting assignments (literature search, team contract, proposal) has also played an important role. Allowing the students time to work with their group throughout the semester gives time for team building and navigating group dynamics before the in-lab work begins. Scheduling proposal submission early enough to order required chemicals is also helpful to maximize lab time.

While research inherently involves uncertainty and open-ended exploration, students benefited from clearly defined milestones, detailed rubrics, and frequent check-ins. Providing examples of paper-based assays, experimental design, and structured feedback (either verbally or in writing) helped reduce ambiguity and improve the quality of both the process and final products. A balance of autonomy and guidance was key to maintaining motivation and progress.

Group size played a significant role in student engagement and productivity. Smaller groups (2–3 students) fostered stronger ownership, accountability, and communication. Larger groups often experienced imbalances in workload and decision-making.

For implementation into larger classes sizes, there are several modifications that would make this experience scalable. First, it would make sense to put bounds on the types of samples or analytes that students could investigate such as metals in water samples or components of beverages. An instructor could also focus on translating solution-phase spectrophotometric or titrimetric analyses from laboratories done earlier in the semester. This would limit the need to purchase additional chemicals. It may also make sense to have a full lab section work on a single assay where groups of students are responsible for determining a single figure of merit. It should be noted that the reliance on cell phones for detection and limited amounts of chemicals is at the core of making this project work with larger, multisection lab courses.

## Supplementary Material





## Notes

Student handouts, notes for instructors, assessment rubrics (PDF, DOCX)
