# Supplementary material for: Development of a Safe, Scalable, Course-Based Undergraduate Research Experience for Analytical Chemistry: The μCURE Project
Source: J Chem Educ. 2025 Aug 15;102(9):4024–32. doi: 10.1021/acs.jchemed.5c00809 (PMC12424185; doi:10.1021/acs.jchemed.5c00809)
Supplement: Supplementary file 2 [file ed5c00809_si_002.docx]

**Supporting Information**

**Development of a Safe, Scalable, Course-Based Undergraduate Research Experience for Analytical Chemistry:  the µCURE Project**

Kimberley A. Frederick^1^*, Maury E. Howard^2^, Kelly Y. Neiles^3^, Daniel F. Scott^4^ and Rebecca A. Hunter^5^*

^1^Department of Chemistry, Skidmore College, Saratoga Springs, NY 12866, United States

^2^Department of Chemistry and Biochemistry, Virginia Wesleyan University, Virginia Beach, Virginia 23455, United States

^3^Department of Chemistry, St. Mary’s College of Maryland, St. Mary’s City, MD 20686, United States

^4^Department of Chemistry, Centre College, Danville, KY 40422, United States

^5^Department of Chemistry, The College of New Jersey, Ewing, NJ 08628, United States

*Corresponding author email addresses: kfreder1@skidmore.edu, hunterr@tcnj.edu

**Table of Contents**

*Student Skill Development Assignments*

[Summary of Assignments](#_Summary_of_Skill)………………………………………………………….. 2

[Concentration Calculations](#_Microfluidics,_Moles,_and)………………………………………………………… 3

[Adapting Procedures from the Literature](#_Adapting_a_Procedure)………………………………………….. 5

[Arguing Conclusions from Data](#_Analytical_Method_Validation)…………………………………………………… 7

*Student CURE Assignments*

[Overall Project Description](#_heading=h.kd4jgx6n1d1q)………………………………………………………… 13

[Project Timeline](#_heading=h.dmycxcpkgjy0)……………………………………………………………………. 14

[Literature Search Activity](#_heading=h.ncv6vte9pt2y)………………………………………………………….. 15

[Group Contract](#_Device_Design_CURE_1)……………………………………………………………………... 17

[Group Literature Search](#_heading=h.ibcuk3oo3gj)……………………………………………………………. 19

[Project Proposal Draft](#_heading=h.uetdrdsp6svk)……………………………………………………………… 21

[Final Proposal](#_heading=h.jumnyvpg3l6g)……………………………………………………………………… 23

[Weekly Progress Reports](#_heading=h.6xumcra4260b)…………………………………………………………... 25

[Final Report](#_heading=h.qsyqhrunifdt)………………………………………………………………………… 27

*Rubrics*

[Literature Search](#_Literature_Search_Rubric)…………………………………………………………………… 30

[Proposal](#_Proposal_Rubric)……………………………………………………………………………. 31

[Weekly Progress Reports/Lab Notebooks](#_Weekly_Report/Lab_Notebook)…………………………………………. 32

[Poster](#_Poster_Rubric)………………………………………………………………………………. 34

[Teamwork](#_Teamwork_Rubric)………………………………………………………………………….. 37

*Other Resources*

[Tutorial Video Links](#_Device_Design_CURE)……………………………………………………………… 38

[Instructor: Experimental Approach Suggestions](#_Instructor:_Experimental_Approach) 39

#### Summary of Skill Development Assignments

Skill development assignments were created to address common points of confusion that were observed during initial testing with students. One assignment focused on how to calculate concentrations of reagents and analytes using a PAD. While concentration calculations are not new to the students, when using a PAD they often need to think about amounts in terms of moles rather than molarity, because the solvent evaporates following deposition onto the device. Most students also had limited prior experience with adapting procedures from the literature, so the instructors implemented a practice opportunity using a provided article and a set of guiding questions. Students were frequently surprised by the effort it took to do this compared to simply following a step-by-step protocol which is common in “cookbook” style laboratories. Throughout the semester, students were also provided with opportunities to design aspects of their own experiments. Each instructor did this to varying degrees by adjusting the level of inquiry of the laboratory experiments they were already implementing. For example, students at TCNJ were required to design their own experiment to determine the concentration of cranberry juice in a cran-apple blend using a spectrophotometer. This experience was early in the semester and was supported with a set of pre-lab questions and a whole class discussion prior to lab work beginning, increasing student confidence in their chosen approach (and the likelihood of a successful experimental outcome). Later in the semester, they did this in a way that more directly related to the CURE with an iron analysis using PADs [[Frederick 2022](https://pubs.acs.org/doi/10.1021/acs.jchemed.2c00370)]. Finally, it was noted during beta testing, as well as a general observation across courses, that students frequently struggle to argue appropriate conclusions from data, particularly when there are many potential data points that can be drawn upon (e.g., analytical figures of merit). As such, an exercise was developed that introduced students to an established argument structure (claim, evidence, and rationale) and walked them through the importance and process of using data to make a decision, rather than relying on preconceived notions or expectations. Each of these assignments is optional depending on what is needed to support the students in both the CURE and the overall outcomes of an individual course.

### Microfluidics, Moles, and Concentration


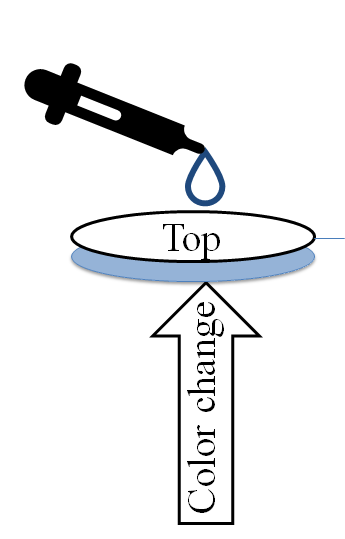
Introduction: Let’s start with a simple paper microfluidic chip – a round circle of paper. Onto that piece of paper, we are going to deposit 50 μL of 100 ppm (w/v) NaCl. As a reminder, ppm (w/v) means mg/L or μg/mL.

50 μL of 100 ppm NaCl

1. Immediately after putting the drop onto the chip, what is the concentration of NaCl?
2. After waiting about 20 minutes, all of the water has evaporated. Describe what is left on the chip.
3. What is the concentration of the NaCl after that 20-minute waiting period?
4. How much NaCl is on the chip after that 20-minute waiting period? (This can be in units of your choosing and there is more than one correct response.)


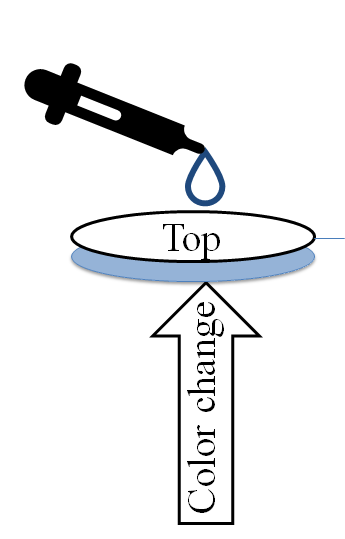
Now, we add 120 μL of sample (some other aqueous solution NOT containing NaCl) onto the same chip.

120 μL of sample

1. After adding this new aqueous solution, how much NaCl is in the solution on the chip? What is the concentration of NaCl? Show any relevant work.
2. In your own words, explain why the concentration has changed.
3. If my analyte is contained in the 120 μL solution that was added second, which of the two concentrations would be most important?
4. In your assay development, you realize that you actually need to increase the concentration of NaCl in the final assay solution without changing the final volume of sample added (120 μL). Describe two ways that you can do that.
5. Explain, in your own words, why the concentration of the initial solution is not really the most important way to think about amounts of reagents when working with microfluidic devices. What might be a better way to express the amount of reagent or analyte deposited onto a microfluidic chip?

We will discuss your answers to this question at the beginning of the lab period.

### Adapting a Procedure from the Literature

**LEARNING OBJECTIVES**

1. Understand the function of an abstract.
2. Identify the chemicals and solutions that would be needed to carry out a laboratory method as described.
3. Describe the key figures of merit used to evaluate a method.

**ASSIGNMENT**

Later in the semester, you will be developing your own procedures for some labs as well as a final project. This exercise is meant to give you an introduction to this process.

The questions below are based on the article **Comparison of Colorimetric Analyses to Determine Cortisol in Human Sweat** by Ethan Tu, Paul Pearlmutter, Michelle Tiangco, Gia Derose, Lina Begdache and Ahyeon Koh***** (*ACS Omega* 2020, 5, 14, 8211–8218, March 31, 2020 <https://doi.org/10.1021/acsomega.0c00498>)

1. Begin by quickly reading through the abstract. What are some key points that you take away from this overview?
2. When we read papers, it’s almost always with a particular goal in mind. For example, you could read a paper to understand a new finding, or you might read a paper to help you to brainstorm solutions to an ongoing research question. For this assignment, we are reading this paper with the goal of implementing the analytical method they describe. Now re-read the abstract with that goal in mind and answer the following questions. Write down as much information as you can but recognize that the abstract is only a summary, so there is more detail to come as we dig into the paper further.
   1. What chemical compound (analyte) are the authors trying to measure?
   2. What is the sample (matrix) that the analyte is in?
   3. What is the method (chemical, instrumental, etc.) being used to measure this analyte?
   4. What are the performance metrics they are using to evaluate the method? (These are almost always related to “figures of merit” and will be discussed further in this course.)
3. The next section to read is Materials and Methods. This is the section that *should* have all or most of the information you need to turn the work in this paper into a working protocol. Please answer the following questions about *only* the blue tetrazolium assay.
   1. What reagents will you need to conduct this assay? (Often there is a paragraph describing the reagents at the beginning of this section. In this case, it is in the Supplemental Information.) Based on the provided information, what do you need for this method and what information do you have about where to get the chemicals and the form that they are in? Summarize this information in a list format.
   2. What solutions will you need to prepare? Again, make a list.
   3. Are there any solutions where you would need to do calculations to prepare it? It is more common than not that you will be given concentrations but not recipes for how to make those solutions. Write out a detailed procedure for anything you need to prepare.
   4. Now let’s look at any *standard solutions* that you will need to prepare. What is the concentration range of the standards? If they don’t provide that information in the Materials and Methods, then you can see if it might be apparent from figures in the Results and Discussion. In this case, figure 2 might be helpful.
   5. How will you make the standards? The most common approach here is to make a more concentrated *stock solution* and then make dilutions of your stock solution into your matrix. What is the matrix in this assay? What concentrated stock solution will you make and how much? How will you dilute from there? Write a detailed procedure.

Please note: There is NO one correct way to do this! There are many reasonable ways so just pick one. If you are required to make way more of a solution than you will ever need (1 L for example!), then you might want to think about doing a serial dilution where you make an intermediate concentration and dilute from there.

- 1. Once you have made the standards, how will you mix them with the other reagents to get the solution you will actually analyze? Again, write a detailed procedure.
  2. Now that your solutions are ready to be analyzed, we need to know what instrument to use and what the conditions are. In this case, you will be using UV-vis absorbance spectroscopy, but we need to see if there are other parameters suggested (e.g., wavelength). Create a table of any instrument settings (these might be found in Materials and Methods or Results and Discussion).

1. The main goal of the Results and Discussion section is to understand the metrics that were used by the author to assess the performance of their assay. Please review the highlighted sections of the Results and Discussion.
   1. What figures of merit did you find and what are their quantitative values?
   2. What figures of merit do you think are missing?
   3. Is there anything else mentioned that would be important as you try to use this method?

### Analytical Method Validation & Arguing Conclusions from Data

**Learning Objectives:**

At the conclusion of this activity, students will be able to…

- explain the importance of method validation.
- identify the type of data required to complete a method validation.
- evaluate analytical figures of merit.
- argue a conclusion based on data from scientific experiments.

**What is analytical method validation?**

Method validation is the process of demonstrating that an analytical method is suitable for its intended use (e.g., can the method accurately and precisely measure the expected concentration of the analyte within a specific sample). The validation process involves conducting a variety of studies to evaluate method performance under defined conditions. Properly designed and successful method validation studies create confidence in the reliability of a test method. Method validation is one of several important quality system components that are designed to ensure the production of scientifically valid and useful analytical data.

The level of validation required is dependent on a number of variables. For example, methods to be used worldwide for medical applications require high precision and accuracy, while analytical methods used for research purposes frequently require less rigorous validation. Typical validation procedures include:

- Quality Control – requires regular analysis of laboratory reagent blanks, laboratory fortified blanks and matrix samples (spiked with analyte); determination of limit of detection
- Accuracy & Precision – acceptable levels depend on application
- Method Performance – requires demonstration of acceptable calibration before, during, and after sample analysis; tests the reproducibility of a method during day-to-day operations

Many United States Government science agencies develop and validate new methods; these methods are typically freely available online. For example, you can find methods from the [EPA](https://www.epa.gov/measurements-modeling/collection-methods) and [NIST](https://www.nist.gov/publications/search?ta%5B0%5D=248421). You can see the FDA method validation guidelines outlined [here](https://drive.google.com/file/d/1xJ529jJahQXBIhXRdx2R8ct_A7zdF6xD/view?usp=sharing). The government also issues [method validation guidance for industry](https://drive.google.com/file/d/1uYGwbuIdaeQeCDxhpNO1-x79lwZ9O4IL/view?usp=sharing) (this example is specific to bioanalytical methods). Private non-profits, like AOAC International (Association of Official Analytical Chemists) also help to develop [official methods of analysis](https://www.aoac.org/official-methods-of-analysis/), as well as conduct certifications of third-party testing laboratories. The U.S. Pharmacopeia (USP) is an independent non-profit that is focused on the development of safe medicines, dietary supplements, and foods; they provide reference standards and testing specifications for these types of products. Another well-known non-governmental organization that develops standards is the [International Organization for Standardization](https://www.iso.org/standards.html) (ISO). Specifically, [ISO 17025](https://www.iso.org/ISO-IEC-17025-testing-and-calibration-laboratories.html) provides general requirements for testing and calibration laboratories.

Method verification is similar to validation but is specifically focused on evaluating a method’s viability in a *new* laboratory environment. For example, if you are a lab manager looking to implement an EPA standard method in your water quality lab, you need to *verify* that you can achieve performance results similar to those detailed in the EPA validation report with your specific instrumentation and technicians. You will likely be verifying a few performance characteristics, such as specificity, repeatability, and limit of detection.

If you are creating a completely new analytical method (which you will be doing later this semester!), it must be fully characterized and validated by determining a variety of **analytical figures of merit**, including:

- **Sensitivity** – the capability of responding reliably and measurably to changes in analyte concentration; quantified as change in signal per unit of concentration (the slope of the calibration curve)
- **Linear/dynamic range** – the range of concentrations in which the instrument/method response is linear
- **Limit of detection** – the smallest quantity of analyte that is “significantly different” from the blank
- **Limit of quantitation** – the smallest amount of analyte that can be measured with reasonable accuracy
- **Selectivity/specificity** – how well a method can identify a specific analyte in a mixture without interference from other components
- **Accuracy** – a measure of how close the results obtained from an analytical method are to the true or accepted value of a substance in a sample; typically determined using a spike recovery
- **Precision** – repeatability of the measurement technique, typically determined using the percent relative standard deviation (%RSD) of replicate measurements

We will discuss these figures of merit further in class.

**PART I. What data do I need to validate a new method?**

For the following section, consider the fluorescence analysis of blood samples for two ovarian cancer biomarkers, human epididymis protein 4 (HE4) and cancer antigen 125 (CA125). When thinking about acceptable validation criteria, also consider the consequences of a test result that incorrectly indicates the presence or absence of a biomarker.

This is meant to be a brainstorming exercise, so **spend no more than 20 minutes answering these questions.**

1. Given the nature of the measurement, what analytical figures of merit and other validation criteria do you think would be most important for being confident in the analytical result?
2. When creating a calibration curve for the instrument (a fluorimeter, in this case) how would you determine what concentration range of standards should be run?
3. When a quality control sample is measured multiple times by the same method, the quantified amount of analyte can vary with each measurement. What might be the cause of these variations? Explain what you think would be an acceptable level of variation.
4. While a calibration curve for this method is usually constructed using standards dissolved in a buffer solution, the spike recovery analysis is typically conducted using the intended sample matrix (e.g., blood). Why is this the case? What would it mean if the percent recovery were very high (>120%) or very low (<80%). How would you attempt to correct for an unacceptable spike recovery result?
5. If a sample was split and tested for these biomarkers using different fluorimeters in the same lab, should the results be identical on each instrument? Briefly explain your reasoning.
6. In a lab where these analyses are conducted, there are a number of trained technicians who may perform the actually analyses. Is it a problem if an analysis does not yield identical results when different technicians analyze the same sample? Briefly explain your reasoning.

**PART II. How do I draw reasonable and meaningful conclusions from all this data?**

The goal of a scientific experiment is to generate data and observations in an attempt to answer a question. Once the data is collected and analyzed, this evidence can be used to construct and support an argument or conclusion. A conclusion is essentially a final judgement that can be made based upon the evidence collected. Creating a well-structured and well-reasoned argument is a skill that takes some practice, especially when drawing upon a large body of evidence, such as multiple analytical figures of merit.

A scientific argument or conclusion has three main parts:

| **CLAIM** | supported by | **EVIDENCE** | and justified using | **RATIONALE** |
| --- | --- | --- | --- | --- |
| *an answer to the original research question* |  | *data or other information that shows trends or relationships* |  | *the reasoning that explains the evidence and why it supports the claim* |

The key to sound scientific argumentation is to make a claim that directly addresses the original research question and is supported with sufficient appropriate evidence. The evidence – which may include measurements, observations, or even findings from other studies – must be appropriately analyzed and interpreted for the claim to be valid. The rationale should connect the evidence to the claim in a succinct and logical manner, and should include an explanation of any assumptions, concepts, or theories underlying the analysis/interpretation of the data.

***Let’s practice…***

Now you’ll have a chance to determine some analytical figures of merit based on provided data and use them to draw conclusions. Below is the plot for the fluorescence detection of two ovarian cancer biomarkers, CA125 and HE4, collected with the same analysis method, using standard solutions prepared in buffer. Use this plot to answer the questions below.


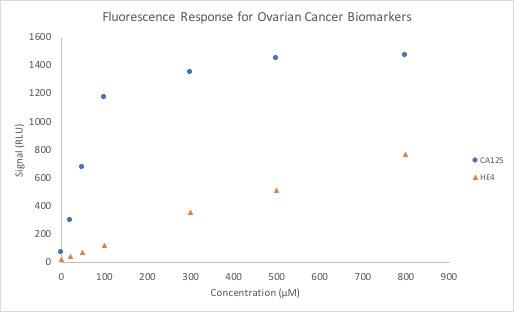


1. For which analyte does the method have the largest (linear) dynamic range? Briefly explain how you determined this.
2. The linear portion each plot can be fit to one of the below equations:

y = 0.9362x + 36.0

y = 11.520x + 36.0

Predict which analyte is described by each line of best fit equation. Briefly explain your prediction.

1. For which analyte is the method most sensitive? What data did you use to determine this?
2. A method blank was analyzed 10 times and generated an average signal of 36.0 ± 8.4 RLU. Calculate the detection limit for each biomarker.
3. A quality control (QC) sample was created by spiking a known concentration of CA125 in blood. A spike recovery of 92% was obtained. Based on this result, is the described external standard calibration method sufficient?
4. If both biomarkers were present in a single sample, would you expect any error in your determined concentration of CA125? Briefly explain.
5. Human serum albumin (HSA) is the most abundant protein in blood. The method has been confirmed to not respond to HSA. On the above plot, draw the response you would expect if the method was run using a series of HSA standard solutions in buffer.
6. Write a short conclusion paragraph addressing…
   1. the feasibility of using the method for analysis of real blood samples, considering that the typical concentration range of these biomarkers is 10–50 µM.
   2. any additional experiments needed if the provided data is not sufficient for validating the method.

Your conclusion should follow the “Claim, Evidence, Reasoning” format outlined above!

# Device Design CURE

*The acronym CURE stands for “Course-Based Undergraduate Research Experience.” Research experience has been demonstrated to have many positive benefits for students in addition to providing an opportunity to hone skills which are highly valued by employers. I strongly believe that all students deserve the benefits of a research experience, and knowing that not all of you will choose to participate in CHE 493, I find it important to provide an experience as part of this course.*

**Project Objective**

Much of analytical chemistry involves careful experimental planning prior to even stepping into the lab. Whether you’re in industry, working for a government agency, or even doing consulting work, a well thought out experimental plan is essential to ensure delivery of a final result that meets accuracy and precision requirements of the given problem. However, accuracy and precision are not always the most important factor to consider in experimental design. Sometimes cost-effective, portable devices that are “just accurate enough” are preferred, especially for field applications.

Paper microfluidic devices are excellent because they are inexpensive to produce and can perform complex analytical operations with minimal technical skill required for the user. Indeed, they meet the World Health Organization’s ASSURED standard (Affordable, Sensitive, Specific, User-friendly, Rapid and Robust, Equipment-free and Deliverable to end-users).^^[[1]](#footnote-1)^^ This technology has been used widely for applications in biomedical diagnostics, environmental analysis, forensics, nutritional supplements and virtually anything else you can imagine.

While some analytical chemists work primarily on optimizing existing methods for use with new types of samples, others work to design brand new measurement techniques. This final research project is meant to give you a sense of this process, including thinking about device design, sample preparation, method characterization/optimization, and data analysis. **Specifically, you will be working on the design of a new paper-based, colorimetric analytical assay. The color change can be detected using a cell phone app, allowing for low cost, rapid quantitative analysis.**

**Learning Outcomes**

*Students will…*

- compare different types of solution-phase colorimetric assays for the same analyte found in the literature.
- propose a device design and experimental plan for the adaption of a solution-phase colorimetric assay to a paper microfluidic platform.
- investigate experimental conditions that allow them to optimize the operation of their paper microfluidic device.
- apply methods of data analysis and calculate the analytical figures of merit of their paper microfluidic device.
- evaluate the analytical figures of merit of their paper microfluidic device.
- demonstrate their ability to productively participate in a team to complete a multi-week project.

# Sample Timeline

| **Week of** | **Task** | **Items Due** |
| --- | --- | --- |
| 27 January | ----------------- | ----------------- |
| 3 February | Intro to project | ----------------- |
| 10 February | ----------------- | Individual Literature Search Assignment |
| 17 February | ----------------- | Group Contracts |
| 24 February | ----------------- | ----------------- |
| 3 March | ----------------- | Group Literature Search Assignment |
| 10 March | ----------------- | Proposal Draft |
| 17 March | SPRING BREAK | |
| 24 March | Proposal revisions and calculations, draft weekly plan – due at end of lab period | |
| 31 March | ----------------- | ----------------- |
| 7 April | Work on projects in lab | ----------------- |
| 14 April | Work on projects in lab | Progress Report 1 |
| 21 April | Work on projects in lab | Progress Report 2 |
| 28 April | Work on projects in lab | Progress Report 3; Poster Draft |
| 5 May | Work on projects in lab -  wrap up & clean up | Progress Report 4; Poster Feedback |
| Finals | ----------------- | Final Poster “Presentations” |

# Device Design CURE – Literature Search Activity

**PROJECT TIMELINE**


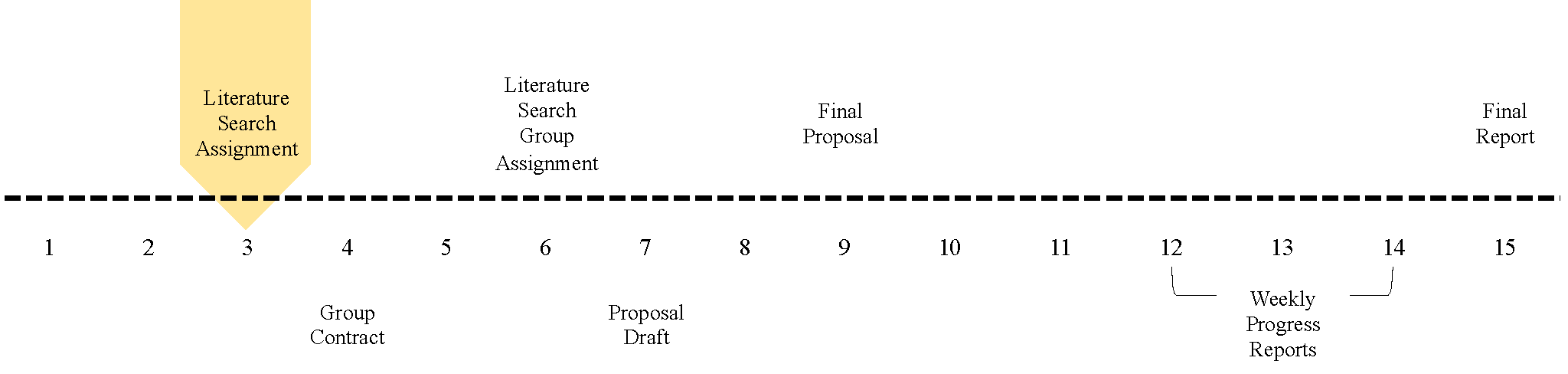


**Assignment Purpose (the *Why*)**

In chemistry fields we generally develop ideas for our experiments not out of thin air, but instead based on the work that has been done previously. The process by which chemists read, interpret, and catalog previous work on a topic area is a learned skill that must be honed with practice. ***The purpose of this assignment is to provide students the opportunity to practice identifying, interpreting, and cataloging previous work on a given topic.***

Upon completing this assignment, students will be able to:

- search the scientific literature for examples of solution-phase colorimetric assays and summarize the most important findings.
- evaluate different types of solution-phase colorimetric assays for the same analyte found in the literature.

**Assignment Description (the *What* and *How*)**

In this assignment, students will search the scientific literature and identify suitable sources to support the development of a paper analytical device (PAD) for the identification and quantification of a target analyte. Appropriate assays will involve some type of solution-based colorimetric (color changing, absorbance, and/or UV-vis) method. Appropriate sources will yield experimental procedures for a target analyte that are potentially adaptable for use in a PAD. The sources may also yield background information on the significance of the target analyte.

Each student should *independently* search the scientific literature and complete the following tasks:

1. **Decide on your analyte.** Think of an analyte you might be interested in measuring, and the sample in which you’d like to measure it. If you are not completely sure on an analyte, you can start with a group of potential analytes or matrices (e.g., hormone disruptors, NSAIDS, type of drugs, environmental contaminants).
2. **Search for articles about the analysis of your analyte and/or sample of interest.** Use internet resources (e.g., Google Scholar, SciFinder) to search the peer-reviewed scientific literature related to the colorimetric assay you are interested in. *Start by searching for a combination of the terms: your analyte + colorimetric + solution + assay.*

Searching for particular articles can be challenging; it will likely take several attempts to find good options. You can change the order and combination of the keywords you are searching and use synonyms (try “quantification” or “detection” instead of “assay”, leave “solution” out, etc.). Once you start to find some useful articles take notice of the titles/keywords and adjust your search as needed.

As you are searching you will likely find some very specialized assays and detection methods that will be difficult to replicate for your project. *Some terms to* ***avoid*** *when you see them as part of the experimental methods or title that will likely make it difficult to replicate the experiment include:* synthesize(d), reflux, nanoparticle (often gold or silver), organic solvents (methanol, ethanol, etc.), live cells, enzymes, chromatography, HPLC. This is not an all-inclusive list but will help you narrow it down. Additionally, you should **avoid** articles where the authors have already adapted the assay to a PAD, as that’s the whole point of this project!

An additional search strategy is to find a colorimetric assay for a particular analyte and then try to adapt this assay for your sample of interest. For example, if you find an assay for lead in water, you may be able to adapt it to measure lead in juice or soda.

1. **Create an annotated bibliography summarizing your search.** Your bibliography must include 5–7 peer-reviewed research articles (not random pages off the internet). At least 5 of these must be a research article (primary source), and 1–2 may be a cited review article (secondary source). In your annotated bibliography, include the following:
   1. A list of keywords that you found most useful for searching. (NOTE: Keeping track of this can save you hours of searching later.)
   2. A bibliography entry for each article which includes:
      1. A citation (ACS format)
      2. The abstract (you can copy/paste this directly from the article)
      3. An annotation that addresses the following:
         1. What were the research objectives and major findings?
         2. What measurements were used to meet the research objectives?
         3. How did the researchers conduct their analysis? (Can their methods be used for your project? What challenges do you think you might run into?)
         4. Why did you select this article over others?
         5. What questions do you have about the article? What was confusing?

Cited sources will be used to a) find information about the target *analyte* you have selected (Why should it be monitored? What levels should you expect to see in typical real-world samples, etc.?); b) identify a standard analytical method you will adapt to make your PAD (*you should seek out at least 3 different methods for the target analyte*); c) find information on the reagents used/needed (purity, vendor, etc.), procedures employed for analysis and any instruments used/needed; and d) find information that will be useful in developing the procedure (and overall experimental design) you will apply and to adapt that procedure to a PAD.

**Additional instructional resources**:

[ACS Guide to Scholarly Communication](https://pubs.acs.org/doi/book/10.1021/acsguide)

**Grading Criteria**

Your grade on this assignment will be determined using the Annotated Bibliography you submit, using the Literature Search rubric, which contains categories from the ELIPSS Information Processing Rubric.

# Device Design CURE – Group Contract

**PROJECT TIMELINE**

**
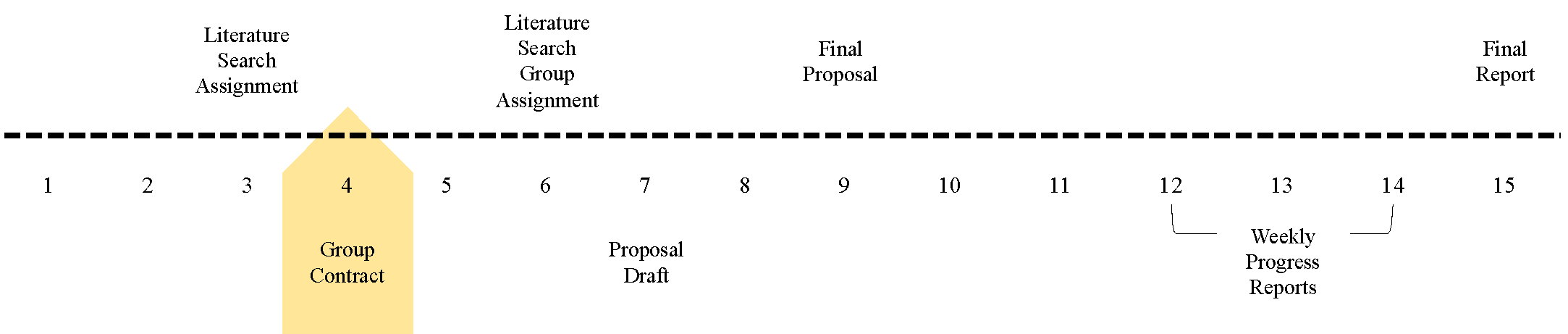
**

**Assignment Purpose (the *Why*)**

Group work refers to learning experiences in which students work together on the same task. Peer interactions can positively affect learning experiences by preparing students for work beyond the classroom. When students work together to solve problems, they are able to construct knowledge together, rather than passively absorbing information. As such, students may learn more effectively working cooperatively in groups as opposed to working alone or in competition with other students. While working collaboratively has its benefits, maintaining a positive and productive work environment can be challenging. When working as a team, work should be thoughtfully planned to enhance everyone’s skills and abilities towards achieving learning outcomes. ***The purpose of this assignment is to have group members begin discussing their expectations for collaborative work on the final lab project.***

Upon completing this assignment, students will be able to:

- reflect on their ability to productively participate in a team to complete a multi-week project.

**Assignment Description (the *What* and *How*)**

In this assignment, each group will complete the form on the next page. You should plan to have a robust discussion about each item as you complete the contract and record your thoughts. This is a living document and can be revisited and amended throughout the semester.

**Grading Criteria**

Your grade on this assignment will be based on completion of the document. At the end of the semester, you will also be evaluated using the ELIPSS teamwork rubric.

**Research Project Group Contract**

Take a moment to introduce yourself to other group members. Record each group member’s answers to the following questions in the boxes below.

| **Questions** | **Responses** | | |
| --- | --- | --- | --- |
| What name and pronouns do you prefer to go by? |  |  |  |
| What is one goal you have for the project? (e.g., something you want to learn or achieve) |  |  |  |
| What are your general research/scientific interests? |  |  |  |
| What strengths do you have that might benefit the lab group as a whole? |  |  |  |
| What is the best way to contact you? Provide any relevant contact info. |  |  |  |

**What are some agreements your group would like to have to ensure an effective working environment? (5–10 is a reasonable number) Some things to consider are provided below.**

- *What are your expectations regarding preparedness for lab each week? What do you want to have ready to go as soon as you arrive to lab?*
- *What are your expectations regarding how the lab time will be spent each week, and what type of work will be done during the lab period vs. in between lab periods (e.g., data analysis)?*
- *When/how do you want to meet outside of the lab period to move the project forward (e.g., data analysis, weekly reports, etc.)?*
- *How do you plan to incorporate multiple viewpoints/ideas when working on the project?*

**Consider possible roles for the members of the group. Some suggestions are provided below, but you may come up with some of your own. Note that all group members are responsible for fully participating in all aspects of the project. How will you rotate roles to ensure this happens?**

- File master**:** *keeps original copies of all documents; manages group notebook*
- Facilitator: *leads discussions during meetings*
- Data analyst: *works up the data for the week*

**How will you support each other if someone isn’t or can’t keep the requirements of the contract? When will you need to notify your instructor? What will you suggest to the instructor if a group member is not meeting their obligations in this contract?**

**Signatures:**

# Device Design CURE – Group Literature Search Activity

**PROJECT TIMELINE**


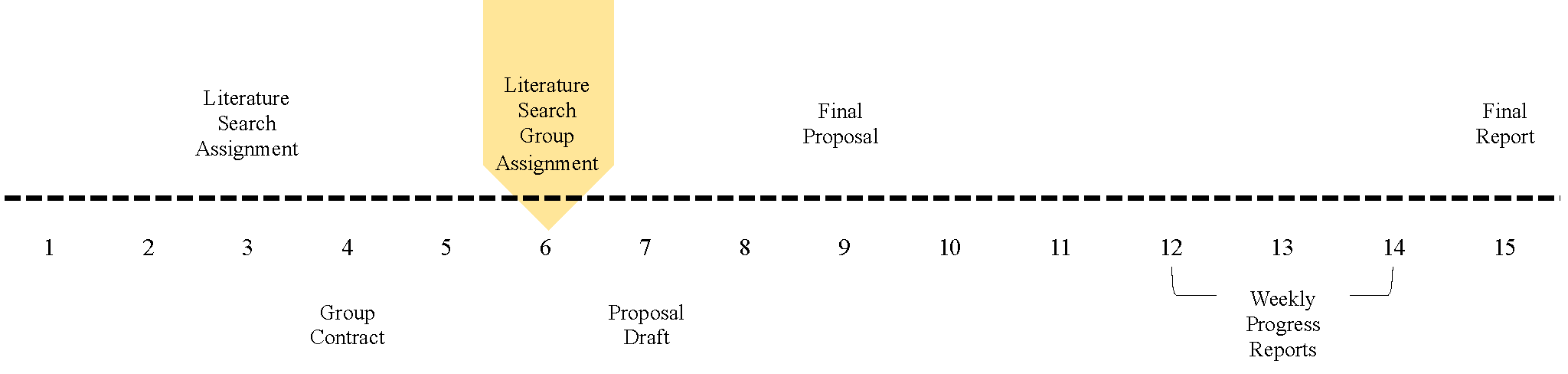


**Assignment Purpose (the *Why*)**

In chemistry fields we generally develop ideas for our experiments not out of thin air, but instead based on the work that has been done previously. The process by which chemists read, interpret, and catalog previous work on a topic area is a learned skill that must be honed with practice. ***The purpose of this assignment is to provide students the opportunity to practice identifying, interpreting, and cataloging previous work on a given topic.***

Upon completing this assignment, students will be able to:

- search the scientific literature for examples of solution-phase colorimetric assays and summarize the most important findings.
- evaluate different types of solution-phase colorimetric assays for the same analyte found in the literature.

**Assignment Description (the *What* and *How*)**

In this assignment, students will search the scientific literature and identify suitable sources to support the development of a paper analytical device (PAD) for the identification and quantification of a target analyte. Appropriate assays will involve some type of solution-based colorimetric (color changing, absorbance, and/or UV-vis) method. Appropriate sources will yield experimental procedures for a target analyte that are potentially adaptable for use in a PAD. The sources may also yield background information on the significance of the target analyte.

As a group, discuss your sample/analyte of interest and literature findings. This discussion can occur outside of lab or during lab time, and can be done asynchronously if necessary. A discussion during the lab period is recommended, as the instructor will be available to answer any questions. Each student should plan to spend about 5 minutes summarizing their annotated bibliography before the group as a whole discusses how to move forward.

1. **Decide on the analyte and sample your group will measure.** Consider all the analytes and sample types discussed, and decide which you’d like to proceed with as a group. How you make this decision is up to you – the group may decide based on what sounds most interesting, or which assay seems most feasible to adapt.
2. **Search for two additional articles about the analysis of your analyte, sample, or assay types to add to a group annotated bibliography.** Based on your group discussion and any instructor feedback, take a slightly deeper dive into the literature and add these additional articles to a group annotated bibliography (that’s 5 articles in total). These articles may come from an additional internet search, or a group member might have already found an article that is relevant to add.
3. **Write an introduction to your group annotated bibliography.** Use your cited sources to write an introductory paragraph for your annotated bibliography. This paragraph should:
   1. Identify the target *analyte* you have selected, describe *why* it should be monitored, and what levels you *expect* to see in typical real-world samples, etc.
   2. Identify at least 2 analytical methods (e.g., colorimetric reactions) you could adapt to make your PAD. (Having more than one potential method for the same target analyte will allow you to have a back-up plan.)

**Grading Criteria**

Your grade on this assignment will be determined using the Annotated Bibliography you submit, using the Literature Search rubric, which contains categories from the ELIPSS Information Processing Rubric.

# Device Design CURE – Project Proposal Draft

**PROJECT TIMELINE**


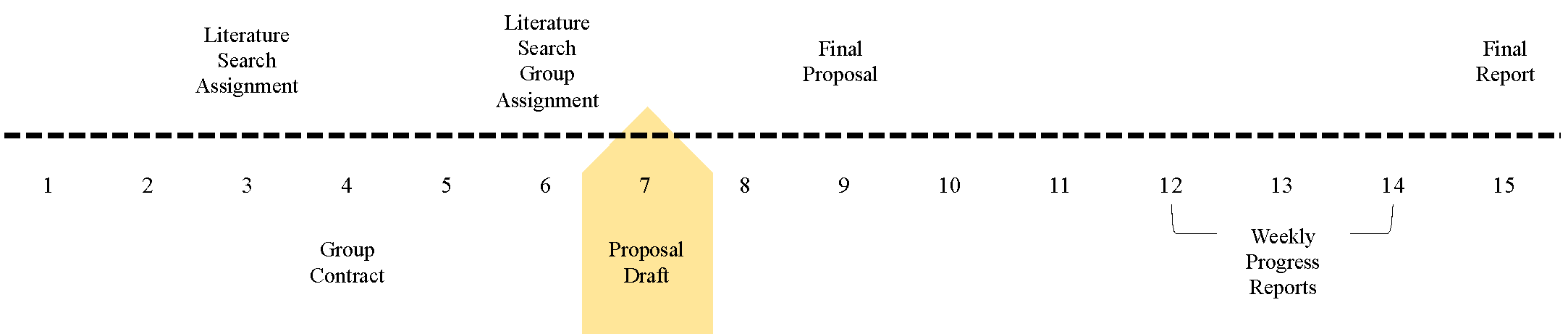


**Assignment Purpose (the *Why*)**

In chemistry fields we often have to utilize previously published methods to create our own experimental procedures. Many students, and early-stage professionals, are surprised to find that this does not simply mean we find a previously published method and use it “as is” in our own laboratories. It is most often the case that the published methods will need to be altered or even combined with methods from other publications to create an experimental process that meets our needs. ***The purpose of this assignment is for students to draft an experimental plan for the development of a PAD based on the peer-reviewed literature. The proposed device should be aimed at measuring a single analyte in a specific type of sample.***

Upon completing this assignment, students will be able to:

- propose a device design and experimental plan for the adaption of a solution-phase colorimetric assay to a paper microfluidic platform.

**Assignment Description (the *What* and *How*)**

In this assignment, student groups will use the literature sources from their annotated bibliography to draft a proposed experimental plan for the development of a new type of PAD. This proposal will identify your sample and analyte of interest, along with the assay you will be adapting and the reagents you require. There is no required length, but a thorough proposal typically spans at least 2 pages. As a group, you should address the below questions in your proposal. The submitted document should provide responses in a numbered list, rather than a narrative, format.

1. Sample and Analyte
   1. What is your analyte?
   2. What is the sample or matrix that the analyte will be in?
   3. What analyte concentration range would you expect in your sample based on the literature? Provide a reference for this information (possibly from your annotated bibliography).
   4. What is the chemistry involved that will provide the desired colorimetric result? You can summarize this in words or draw out the reaction scheme
2. How has this analyte previously been analyzed by others? Briefly summarize at least 2 articles from your annotated bibliography.
3. Figures of Merit
   1. What types of standard(s)/calibration will you use?
   2. What linear concentration range do you expect for your assay based on previously published results? Provide a reference for this information.
   3. What are some likely matrix interferents (present in your sample)? Explain why you are concerned about these specific interferents. Provide a reference.
   4. What specific figures of merit will be important for optimizing and validating your device?
4. Protocol
   1. Will you be developing a [bubble chip](https://youtu.be/BGpUQQ0rSAA), a [flow-through](https://youtu.be/LRwoSCOF7Mk?si=TQa8HRW-DSx4VLjB) or [lateral flow](https://youtu.be/hjiniUa5BHs?si=WPfiUfpRYS2bJohz) device, or some other configuration? If you’re not sure, discuss these options with your instructor.
   2. What type of sample pre-treatment might be required? Why?
   3. How will you validate your method to be sure you’re getting accurate and precise results?
      1. What mock samples will you need and how will you make them?
      2. What controls will you need?
      3. How many replicate samples/standards/calibrations will you run to minimize uncertainty?
   4. How will you collect and analyze your data? Be sure to include what statistical analyses you plan to conduct.
   5. How will you ensure your protocol is robust? Method robustness refers to the ability of an analytical method to remain unaffected by small variations in method parameters (e.g., sample composition, environmental factors).
5. Create a flowchart that summarizes your proposed experimental process. This should be a visual depiction of how you will execute your experimental plan. You will update this as your experiments progress so you can compare your initial plan to what you actually did to create a functional device!
6. Include a list summarizing **ALL** chemicals and supplies you will need.
   1. Include approximate quantities (e.g., concentration and volumes of solutions, grams of solids). Note that solutions will have to be prepared from appropriate solids and solvents and will not be purchased.
   2. Include a link to where this item might be purchased. The best places to look are [Sigma Aldrich](https://www.sigmaaldrich.com/US/en) and [Fisher Scientific](https://www.fishersci.com/). If you can’t find an item here, please consult with your instructor.
7. Complete a RAMP safety analysis based on your proposed protocol.

**Grading Criteria**

Your grade on this assignment will be based only on completion. You will receive feedback on these proposals during the next week and then have an opportunity to make any required changes. Your work will be evaluated using the Proposal Rubric, which contains categories from two ELIPSS Rubrics: problem solving and planning/carrying out investigations.

# Device Design CURE – Final Proposal

**PROJECT TIMELINE**


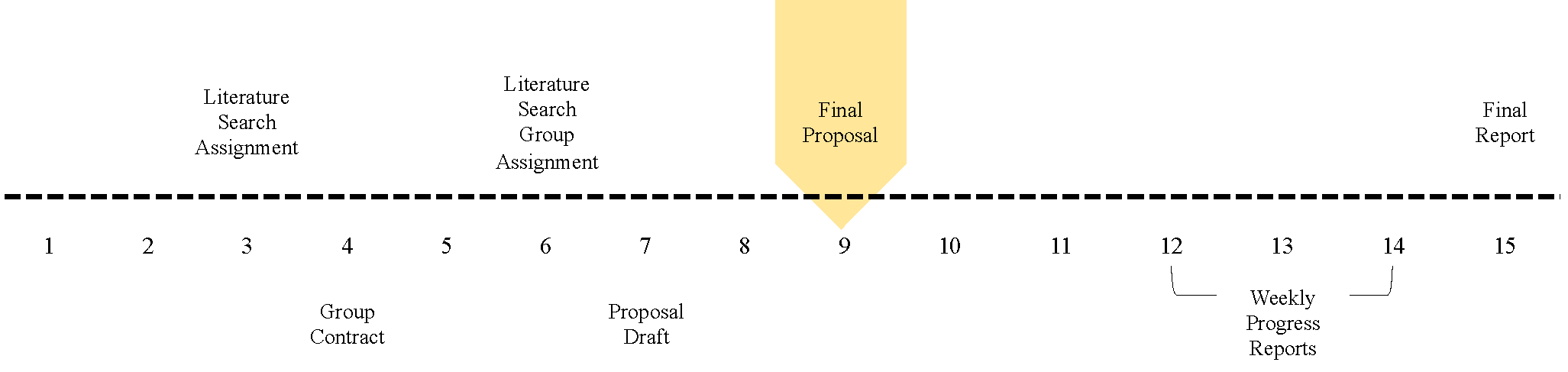


**Assignment Purpose (the *Why*)**

In chemistry fields we often have to utilize previously published methods to create our own experimental procedures. Many students, and early-stage professionals, are surprised to find that this does not simply mean we find a previously published method and use it “as is” in our own laboratories. It is most often the case that the published methods will need to be altered or even combined with methods from other publications to create an experimental process that meets our needs. ***The purpose of this assignment is for students to finalize an experimental plan for the development of a PAD based on the peer-reviewed literature.***

Upon completing this assignment, students will be able to:

- propose a device design and experimental plan for the adaption of a solution-phase colorimetric assay to a paper microfluidic platform.

**Assignment Description (the *What* and *How*)**

Refer to the [Proposal Draft assignment](#_heading=h.uetdrdsp6svk) for all the details. After receiving feedback on your draft, each group should do the following:

**Edit your proposal to address all feedback received.** You many use the same format as before, just edit or add content as needed. Update your RAMP analysis if needed, as well.

**Write a “Response to Reviewers.”** This is standard practice when a draft publication has potential but is not ready for final acceptance. A response to reviewers specifies how the authors addressed each comment the reviewers made and is usually organized by presenting the reviewer’s comments one by one, followed by the authors’ response. You should distinguish your responses from the reviewer’s feedback by using phrases such as “author response” and/or a different font color. Then, each response should clearly explain the change made, or justify why a change was not made.

**Complete all calculations** needed for the preparation of solutions (stock, standards, buffers, etc.) so you are ready to go once lab work begins. Include a table with these details (e.g., identity, solute mass, solvent volume, final concentration).

**Set weekly goals.** Write out at least 2 goals you hope to achieve during the first week of lab work.

**Grading Criteria**

Your grade on this assignment will be based only how thorough and reasonable your experimental plan is. Your work will be evaluated using the Proposal Rubric, which contains categories from two ELIPSS Rubrics: problem solving and planning/carrying out investigations.

# Device Design CURE – Weekly Progress Reports

**PROJECT TIMELINE**


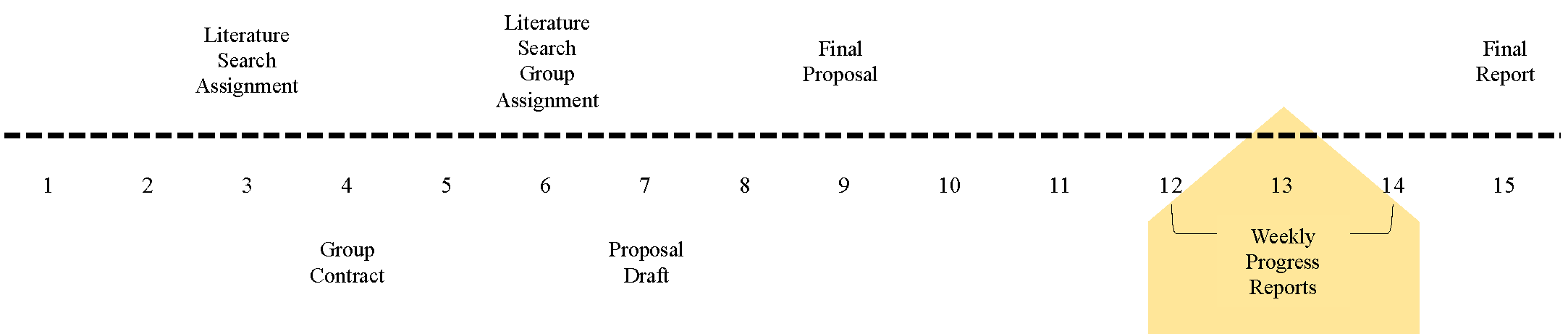


**Assignment Purpose (the *Why*)**

Even with extensive planning, your time in lab will go by quickly. In order to develop and systematically optimize a new PAD, you will need to analyze data along the way and adjust your experimental plan accordingly. It is imperative to do this prior to coming to lab to avoid wasting time. As a bonus, you’ll already have some figures created for the final report! ***The purpose of this assignment is for students to reflect on their experimental goals and findings each week, allowing for adaptation of the experimental plan, as necessary.***

Upon completing this assignment, students will be able to:

- investigate experimental conditions that allow them to optimize the operation of their paper microfluidic device.
- apply methods of data analysis and calculate the analytical figures of merit of their paper microfluidic device.

**Assignment Description (the *What* and *How*)**

During the CURE, each group will submit a weekly report that summarizes progress/challenges from the previous week and plans for the upcoming weeks. These are due ~48 hr prior to the next lab meeting. In these reports, your group will need to address the following (note that questions 4, 5, and 7 are not required for the first report submission):

1. What were your experimental goals this week? Write a short paragraph describing what you did to work toward those goals this week.
2. What worked well this week? What can you learn from this? How does this impact your next steps?
3. What didn’t go as expected this week? What can you learn from this? How does this impact your next steps?
4. (Only required for reports 2–4) Work up and data you have acquired so far and provide the calculations. Provide an appropriately formatted figure (e.g., calibration curve) or table to represent the data you have collected so far. NOTE: This figure should not include raw data (e.g., individual color values)! What conclusions can you come to with the data you have required thus far? What data do you still need to collect to form a solid conclusion?
5. (Only required for reports 2–4) Review the data you have worked up thus far, as well as the figure below. What figures of merit can you currently report for your PAD? What additional data do you still need to collect to more fully develop and/or validate the method?



1. Write a short paragraph describing what you plan to try next week. Be sure to **justify** your plan using your experimental goals or lessons learned from the past week.
2. (Only required for reports 2–4) Revisit the experimental flowchart you created as part of your initial proposal. Adjust the flowchart as necessary to reflect what you have actually done/are planning to do. This might mean reordering some items or adjusting/adding arrows to indicate tasks that needed to be revisited/repeated.
3. In what way can the instructor assist in helping you meet your experimental goals for this week?

**Grading Criteria**

Your grade on this assignment will be based on how thorough and reasonable your progress and thought process is each week. Your work will be evaluated using the Weekly Report/Lab Notebook Rubric, which contains categories from two ELIPSS Rubrics: information processing and planning/carrying out investigations.

# Device Design CURE – Final Poster

**PROJECT TIMELINE**


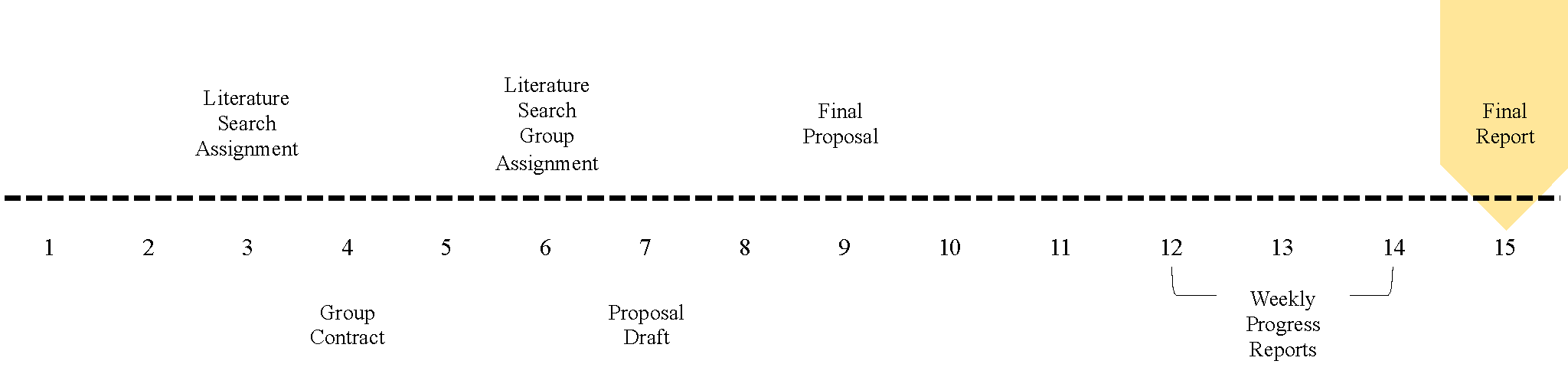


**Assignment Purpose (the *Why*)**

After spending weeks in the lab running experiments, you have likely done a lot of trial and error and accumulated a significant amount of data; hopefully this is all nicely organized in your electronic laboratory notebook and associated spreadsheets. However, raw data in a spreadsheet isn’t a very helpful way to present results. It is up to you to analyze the data, look for trends, find connections, and draw appropriate conclusions from the evidence that was collected, and you’ve already done some of this work each week. Presenting your experimental approach and data in a cohesive way that tells a story is incredibly important for relating this information to the scientific community to be built upon even further. This can be done using more formal writing (reports and journal articles) or in an oral format (posters and slide presentations). ***The purpose of this assignment is for students to present their experimental process and most important and/or exciting results in the form of a poster.***

Upon completing this assignment, students will be able to:

- investigate experimental conditions that allow them to optimize the operation of their paper microfluidic device.
- apply methods of data analysis and calculate the analytical figures of merit of their paper microfluidic device.
- evaluate the analytical figures of merit of their paper microfluidic device.

**Assignment Description (the *What* and *How*)**

Each group will create a poster to present their work. A scientific poster is meant to tell a story about the work that was done and highlight the most important results. It should be visually interesting, as well, rather than containing large blocks of text. Here are some sections you should consider including in your poster (although you are welcome to label them differently):

- Introduction – Provide relevant background information and/or context for your work. Why should others care about your research questions and findings (what makes your work important and/or interesting)?
- Objective or Research Question(s) – What were you aiming to achieve with this work? What questions were you trying to answer?
- Experimental Method – How did you go about answering your research question(s)? Given that this is a method development project, this section may take up a substantial portion of your poster. It should include the following:
  - A written summary of the most important experimental details
  - A visual representation of your method development process – this should be the final version of the flowchart you have been revising throughout the process!
  - Also consider including visuals that show how the device works, including setup and any relevant chemical reactions.
- Results – This section should highlight your most important findings, mostly using figures, tables, and appropriate captions (rather than large blocks of text). Only worked up data should be included in this section (i.e., copy/pasting raw RGB values from a spreadsheet would generally not be appropriate).
- Conclusions – What claims can be made based on the evidence you collected? Be careful not to make sweeping declarations that are not supported by your data.
- Future Work - What questions remain? If you had more time, what additional work would you like to do to improve, build upon, or clarify your results?
- You **must** also include a QR code that links to your electronic lab notebook (be sure the sharing setting allows for instructor access).

This poster can be created using PowerPoint or other illustration tools such as [Canva](https://www.canva.com/). Google Slides is not recommended. Here are some example posters you may find helpful:

- [PAD Method Development](https://drive.google.com/file/d/15qgBm9xBr5j454NIyKo-6Nhd6DMxUhMO/view?usp=drive_link) – This example comes from my CHE 410 course. The [“Better Poster”](https://www.youtube.com/watch?v=SYk29tnxASs&ab_channel=%23BetterScience) format is meant to highlight the most important findings/claim and works well if you collected robust data and can make a strong conclusion.
- [GC-MS Method Development](https://drive.google.com/file/d/1ZCemfeH2C_MWBYcYyYQ5q40UfIEsfCFl/view?usp=drive_link) – This is an example from my research lab. You can see that much of the poster is dedicated to showing how the instrument and sample preparation work, and the parameters that were optimized along the way.
- [Cortisol in Urine Method Development](https://drive.google.com/file/d/1pdGUVJ74RajzmvXTW8Mux1bUieU2qZBN/view?usp=sharing) – Again, you can see the entire center of the poster focuses on conditions that were optimized as part of the method development process.
- [Chromatography Method Development](https://drive.google.com/file/d/1aH_VJ5wzlkPvUSshlvxmjRia2AJEc88X/view?usp=sharing) – These posters were created by a column manufacturer for researchers to display in their labs (so they are not traditional research posters) but they contain method development flowcharts and tips/recommendations for method optimization.
- [COVID Lateral Flow Assays](https://www.compoundchem.com/2021/11/06/lateral-flow-tests/) – The Compound Chem website has some excellent examples of how to summarize information in a visually appealing infographic format.

In addition to physically creating the poster, each group is required to “present” their poster using a voice recording. This presentation should be 5 minutes in length and summarize the poster as if you were presenting it in person at a poster session. While not every student is required to have a speaking role in the recording, all students in the group should contribute to the presentation script. There are multiple ways to record a voice memo depending on your device – here are some instructions for an [iPhone](https://www.theverge.com/23328768/record-voice-notes-iphone-how-to), [Android](https://www.businessinsider.com/guides/tech/how-to-record-audio-on-android), or laptop ([Mac](https://support.apple.com/guide/voice-memos/record-vmaa4b813415/mac) and [PC](https://support.microsoft.com/en-us/windows/how-to-use-voice-recorder-6fbb53d5-0539-abda-a9a4-0bcb84a778e7)). You should save your poster as a PDF and upload it along with your audio file with your final submission in Canvas.

Your poster presentation will be shared with other undergraduates enrolled in an analytical chemistry course as part of an asynchronous poster session. As you interact with the other posters, you will leave the following feedback: 1) one positive comment about what the authors have presented/done particularly well, and 2) one content question that they will respond to.

**Grading Criteria**

You will be assessed on the overall quality of your final poster, in addition to your entire research *process*, which will be documented in your poster as well as throughout your lab notebook pages. Things I’m looking for include:

- the extent to which you were able to combine information from the literature and your prior lab experience with paper devices to generate a new idea
- correct use of prior knowledge in the context of solving a new problem
- clear evidence of logical thought process in your plan
- reasonableness of final experimental approach
- quality of plan execution, including troubleshooting and adapting, as needed.

Your work will be evaluated using the Poster Rubric, which contains categories from two ELIPSS Rubrics: problem solving and critical thinking.

### Literature Search Rubric

| **Category** | **0** | **1** | **2** | **3** | **4** | **5** |
| --- | --- | --- | --- | --- | --- | --- |
| **INFORMATION PROCESSING** | | | ***Evaluating, interpreting, and manipulating/transforming information from the published literature.*** | | | |
| **Evaluating** |  | **Minimally** determined the significance or relevance of information/data needed for the task |  | **Partially** determined the significance or relevance of information/data needed for the task |  | **Completely** determined the significance or relevance of information/data needed for the task |
| **Interpreting** |  | **Inaccurately** provided meaning to data, made inferences and predictions from data, or extracted patterns from data |  | Provided meaning to data, made inferences and predictions from data, or extracted patterns from data **with some errors** |  | **Accurately** provided meaning to data, made inferences and predictions from data, or extracted patterns from data |
| **Manipulating or**  **Transforming**  **(Extent)** |  | **Minimally** converted information/data from one form to another |  | **Partially** converted information/data from one form to another |  | **Completely** converted information/data from one form to another |
| **Manipulating or**  **Transforming (Accuracy)** |  | **Inaccurately** converted information/data from one form to another |  | Converted information/data from one form to another **with some errors** |  | **Accurately** converted information/data from one form to another |

### Proposal Rubric

| **Category** | **0** | **1** | **2** | **3** | **4** | **5** |
| --- | --- | --- | --- | --- | --- | --- |
| **PROBLEM SOLVING** | | | ***Analyzing a complex problem or situation and  developing a viable strategy to address it.*** | | | |
| **Analyzing the Situation** |  | **Minimally** determined the scope and complexity of the problem. |  | **Partially** determined the scope and complexity of the problem. |  | **Completely** determined the scope and complexity of the problem. |
| **Identifying** |  | **Minimally** determined the information, tools, and resources necessary to solve the problem. |  | **Partially** determined the information, tools, and resources necessary to solve the problem. |  | **Completely** determined the information, tools, and resources necessary to solve the problem. |
| **Strategizing** |  | **Minimally** developed a process (series of steps) to arrive at a solution. |  | **Partially** developed a process (series of steps) to arrive at a solution. |  | **Completely** developed a process (series of steps) to arrive at a solution. |
| **PLANNING & CARRYING OUT INVESTIGATIONS** | | | ***Planning and carrying out systematic investigations to test a hypothesis, answer a question, or test solutions that provide evidence in the form of data.*** | | | |
| **Developing a scientifically sound plan** |  | **Minimally** used scientific ideas and theories to plan an investigation to test a hypothesis, answer a question, or test solutions. |  | **Partially** used scientific ideas and theories to plan an investigation to test a hypothesis, answer a question, or test solutions. |  | **Correctly** used scientific ideas and theories to plan an investigation to test a hypothesis, answer a question, or test solutions. |
| **Planning for data collection** |  | **Did not** identify and/or justify the data that needs to be collected. |  | **Partially** identified and justified the data to be collected. |  | **Identified and justified** the data to be collected, along with any limitations. |
| **Developing an executable plan** |  | **Did not** take practical aspects and limitations into consideration in the planning of the investigation. |  | **Partially** took practical aspects and limitations into consideration in the planning of the investigation. |  | **Completely** took practical aspects and limitations into consideration when drafting the experimental procedure. |
| **Developing a safe plan** |  | Took **none** of the safety protocols into consideration while planning the investigation to complete a RAMP analysis. |  | Took **some** safety protocols into consideration while planning the investigation to complete a RAMP analysis. |  | Took **all** the safety protocols into consideration while planning the investigation to complete a RAMP analysis. |

### Weekly Report/Lab Notebook Rubric

| **Category** | **0** | **1** | **2** | **3** | **4** | **5** |
| --- | --- | --- | --- | --- | --- | --- |
| **INFORMATION PROCESSING** | | | ***Evaluating, interpreting, and manipulating/transforming your data.*** | | | |
| **(A)**  **Evaluating** |  | **Minimally** determined the significance or relevance of information/data needed for the task |  | **Partially** determined the significance or relevance of information/data needed for the task |  | **Completely** determined the significance or relevance of information/data needed for the task |
| **(B)**  **Interpreting** |  | **Inaccurately** provided meaning to data, made inferences and predictions from data, or extracted patterns from data |  | Provided meaning to data, made inferences and predictions from data, or extracted patterns from data **with some errors** |  | **Accurately** provided meaning to data, made inferences and predictions from data, or extracted patterns from data |
| **(C)**  **Manipulating or**  **Transforming**  **(Extent)** |  | **Minimally** converted information/data from one form to another |  | **Partially** converted information/data from one form to another |  | **Completely** converted information/data from one form to another |
| **(D)**  **Manipulating or**  **Transforming (Accuracy)** |  | **Inaccurately** converted information/data from one form to another |  | Converted information/data from one form to another **with some errors** |  | **Accurately** converted information/data from one form to another |
| **PLANNING & CARRYING OUT INVESTIGATIONS** | | | ***Planning and carrying out systematic investigations to test a hypothesis, answer a question, or test solutions that provide evidence in the form of data.*** | | | |
| **Developing a scientifically sound plan** |  | **Minimally** used scientific ideas and theories to plan an investigation to test a hypothesis, answer a question, or test solutions. |  | **Partially** used scientific ideas and theories to plan an investigation to test a hypothesis, answer a question, or test solutions. |  | **Correctly** used scientific ideas and theories to plan an investigation to test a hypothesis, answer a question, or test solutions. |
| **Planning for data collection** |  | **Did not** identify and/or justify the data that needs to be collected. |  | **Partially** identified and justified the data to be collected. |  | **Identified and justified** the data to be collected, along with any limitations. |
| **Developing an executable plan** |  | **Did not** take practical aspects and limitations into consideration in the planning of the investigation. |  | **Partially** took practical aspects and limitations into consideration in the planning of the investigation. |  | **Completely** took practical aspects and limitations into consideration when drafting the experimental procedure. |
| **Developing a safe plan** |  | Took **none** the safety protocols into consideration while planning the investigation to complete a RAMP analysis. |  | Took **some** the safety protocols into consideration while planning the investigation to complete a RAMP analysis. |  | Took **all** the safety protocols into consideration while planning the investigation to complete a RAMP analysis. |

#### Poster Rubric

| **Category** | **0** | **1** | **2** | **3** | **4** | **5** |
| --- | --- | --- | --- | --- | --- | --- |
| **PROBLEM SOLVING** | | | ***Analyzing a complex problem or situation and  developing a viable strategy to address it.*** | | | |
| **Analyzing the Situation** |  | **Minimally** determined the scope and complexity of the problem. |  | **Partially** determined the scope and complexity of the problem. |  | **Completely** determined the scope and complexity of the problem. |
| **Validating** |  | **Minimally** judged the reasonableness and completeness of the proposed strategy or solution. |  | **Partially** judged the reasonableness and completeness of the proposed strategy or solution. |  | **Completely** judged the reasonableness and completeness of the proposed strategy or solution. |
| **Executing** |  | **Minimally** implemented the strategy effectively. |  | **Partially** implemented the strategy effectively. |  | **Completely** implemented the strategy effectively. |
| **CRITICAL THINKING** | | | ***Forming and argument or reaching a conclusion supported with evidence by evaluating, analyzing, and/or synthesizing relevant information.*** | | | |
| **Identifying the Aim/Goal** |  | **Minimally** determined the purpose/context of the argument or conclusion that needed to be made |  | **Partially** determined the purpose/context of the argument or conclusion that needed to be made |  | **Completely** determined the purpose/context of the argument or conclusion that needed to be made |
| **Evaluating** |  | **Minimally** determined the relevance and reliability of information that might be used to support a conclusion or argument |  | **Partially** determined the relevance and reliability of information that might be used to support a conclusion or argument |  | **Completely** determined the relevance and reliability of information that might be used to support a conclusion or argument |
| **Analyzing** |  | **Inaccurately** interpreted information to determine meaning and to extract relevant evidence |  | Interpreted information to determine meaning and to extract relevant evidence **with some errors** |  | **Accurately** interpreted information to determine meaning and to extract relevant evidence |
| **Synthesizing** |  | **Inaccurately** connected or integrated information to support an argument or reach a conclusion |  | Connected or integrated information to support an argument or reach a conclusion **with some errors** |  | **Accurately** connected or integrated information to support an argument or reach a conclusion |
| **Forming**  **Arguments**  **(Structure)** |  | Made an argument that only **minimally** included a claim (a position), supporting information, and reasoning |  | Made an argument that p**artially** included a claim (a position), supporting information, and reasoning |  | Made an argument that **completely** included a claim (a position), supporting information, and reasoning |
| **Forming**  **Arguments**  **(Validity)** |  | The claim, evidence, and reasoning were **minimally** logical and consistent with broadly accepted principles |  | The claim, evidence, and reasoning were **partially** logical and consistent with broadly accepted principles |  | The claim, evidence, and reasoning were **completely** logical and consistent with broadly accepted principles |

#### Teamwork Rubric

| ***Interacting with others and building on each other’s individual strengths and skills, working toward a common goal.*** | | | | | | |
| --- | --- | --- | --- | --- | --- | --- |
| **Category** | **0** | **1** | **2** | **3** | **4** | **5** |
| **Interacting** |  | **Rarely** communicated with each other and worked together |  | **Sometimes** communicated with each other and worked together |  | **Consistently** communicated with each other and worked together |
| **Contributing** |  | **Rarely** considered the contributions, strengths and skills  of all team members |  | **Sometimes** considered the contributions, strengths and skills  of all team members |  | **Consistently** considered the contributions, strengths and skills  of all team members |
| **Progressing** |  | **Rarely** moved forward towards a common goal |  | **Sometimes** moved forward towards a common goal |  | **Consistently** moved forward towards a common goal |
| **Building Community** |  | **Rarely** acted as a cohesive unit that supported and included all team members |  | **Sometimes** acted as a cohesive unit that supported and included all team members |  | **Consistently** acted as a cohesive unit that supported and included all team members |

# Device Design CURE – Tutorial Videos

**Fabricating µPADs – Wax Printing**

<https://youtu.be/9GR40jtSmDw>

This video demonstrates how to create devices with hydrophobic barriers made from wax, including the printing and baking steps.

**Fabricating µPADs – Laser Printing**

<https://youtu.be/JDQ8HLxZJOo>

This video demonstrates how to create devices with hydrophobic barriers made from laser printed ink, including the printing and baking steps.

**Fabricating µPADs – Sharpies for Creating Hydrophobic Barriers**

<https://youtu.be/A9Xv58SEXwA>

This video demonstrates how to create devices with hydrophobic barriers using a Sharpie and craft plotter/cutter.

**Fabricating µPADs - Creating Hydrophobic Barriers**

<https://youtu.be/76RFnz3z-qk>

This video describes how to bake your paper-based devices to create hydrophobic compartments/channels to which you will add chemical reagents and samples

**Collecting Data from µPADs - Setup for Image Acquisition**

<https://youtu.be/05T7W4MpjpY>

This video describes how to acquire images with consistent lighting and camera placement to allow for reproducible data.

**Collecting Data from µPADs - Software for Image Acquisition**

<https://youtu.be/msefaOJSHsw>

This video describes how to acquire images with consistent lighting to allow for reproducible data.

**Searching the Literature**

<https://youtu.be/H02PAWHPm2g>

This video discusses tips for searching the literature (using CAS SciFinder) to find solution phase colorimetric assays to adapt for paper-based device development.

**Citing Sources**

<https://youtu.be/2WpA25hljcA>

This video discusses how to create citations from results generated using CAS SciFinder, as well as how to use ZoteroBib. Note: some of this video is specific to students at Virginia Wesleyan University.

# Instructor: Experimental Approach Suggestions

Instructor Guide to help students develop their approach to identify/quantify a target (analyte) using an aqueous colorimetric assay.  May be used with *Micro CURE Check-Off Sheet*.

Info needed (literature):

List of reagents required

Reaction(s) resulting in physical change (complex formation, ppt, etc)

Role of each reagent in reaction scheme (oxidant/reductant, buffer, ligand, etc.)

Procedure (validation):  Initially, ***validate method*** by reproducing the solution-phase reactions.

*Note to instructor:  Some students may skip this step, but for many this requires a closer look at the literature (experimental procedures) to ensure they are able to reproduce a known result.  Then, they can recognize success as they adapt it to paper.* ***Often a quick, small-scale test is sufficient.***

*Questions they may need to consider to get the solution test to work (from the referenced procedure).  Some students overlook key aspects about the reagents required.*

*What are the concentrations of each reagent?*

*Does the order of reagent combination matter?*

*Is there a pH dependence? (buffer used)*

*Is there a redox dependence? (oxidant/reductant used)*

*Is there a time dependence?  (wait time before measurement to ensure full color development)*

*Is there a temperature dependence? (heated or cooled - does this impact development time?)*

*Are any of the reagents light-sensitive? (stored in the dark)*

Adapting the method to PADs:

Type of device considerations:

Are all reagents compatible to be “pre-mixed”?

If not, is a segmented chip a viable option vs. flow-through device?

Is a lateral flow device a good option?

*Possible to conduct multiple tests:  Preliminary testing can utilize ½” punch-outs of chromatography paper, then move to device of choice.*

1. Color / ppt formation on PADs
   1. Reagent concentration (typically needs to be higher than for solutions -- but RELATIVE concentrations should be maintained)
      1. Drying studies (½” disk): pre-mixed? Or mixed on disk? *then dried*
         1. If successful, single chip design should work (*still must be optimized*)
         2. not successful, follow-up with 2 (*next*)
      2. Drying studies (device):
         1. Separate layers for flow through
         2. Separate sections for segmented chip
         3. Separate zones for lateral flow
      3. Color development (*optimize conditions*)
         1. Buffer-pH study (best pH?),
         2. Time testing (shortest time to stability),
         3. Temperature (best temp for dev time),
         4. Light/dark, etc (drying / reaction conditions / storage).
   2. Color Channel (and App) selection (Color Assist, ImageJ, etc.)
      1. Shows a trend that is linear with concentration of ANALYTE
      2. Steeper slope is better for sensitivity  (*Negative slopes are common, so don’t let that throw you off!*
      3. R^2^ closer to 1 is better for precision/accuracy
2. Characterizing the device (FoM): Linearity, linear range, limit of detection, precision, accuracy, sensitivity, selectivity.

*Instructor_Micro CURE Check-Off Sheet MASTER may be used to accompany this guideline.*

*Instructors may have students* develop a flow chart *for planning their experimental approach that they will modify/update as they progress through the project to reflect their actual approach (typically more iterative than they anticipate).  Updated with each weekly progress report.*

**Example: student flow chart of experimental approach (*end of project*):**


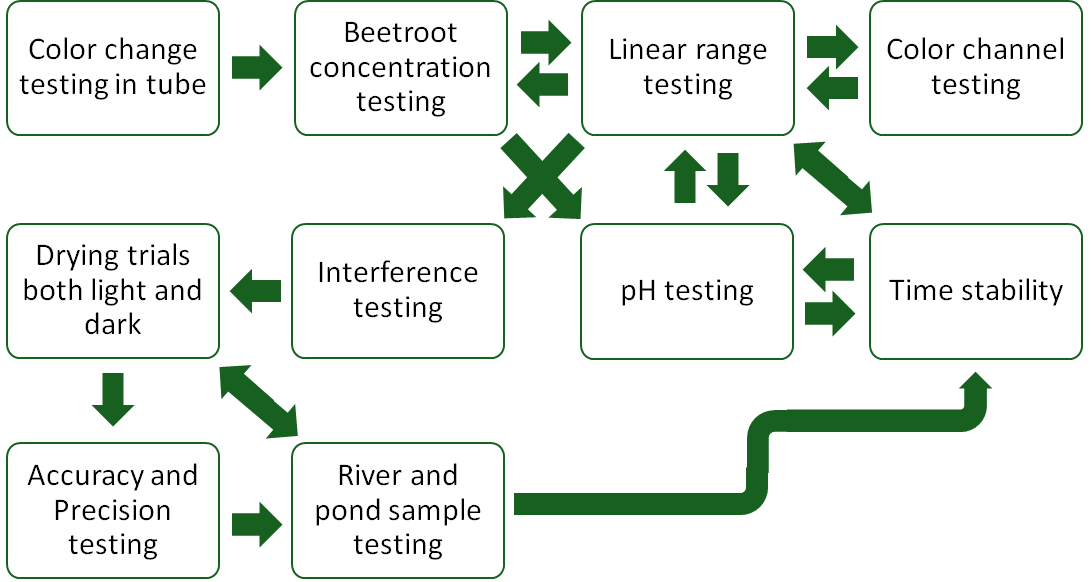


**Tips for MICROCUREs for Instructors**

- Order chemicals early to be sure students have what they need when lab time for experiments begins.
- Be transparent about the iterative process of science and the likely failure, repeating, and modifying of experiments.
- The µPADs allow reactions to happen and be visualized quickly. Be prepared to try multiple iterations of an experiment in a single day.
- Consider using a box or paper cup to control the external lighting in the room when taking pictures of the devices. External light can drastically alter the intensity from run to run.
- Make sure students come with an experimental procedure before the day’s lab. A lot time can be wasted planning out what to try in the lab if the students are not prepared.
- ImageJ can be finicky when downloading to Macs. You have to allow the software to be installed from an untrusted source.
- Start with a wide concentration range of standard solutions, the solutions based assays don’t always translate well concentration wise to the paper based assays.
- When baking the chips, cut the sheets into smaller sections to help with even baking. If chips are underbaked, you can place back in the oven, but will likely need additional time to heat the wax back up.
- Students may not get to a fully functional paper-based assay. Reassure students they are graded on the scientific process and not a perfect paper-based assay.
- Plot all of the color channels to start, and select the best channel from the visualized responses.
- Students may start by characterizing their device design for appropriate volumes. Mixing colored solutions (dyes or food coloring in water) and quantifying color intensity is a good way to get familiar with their set up.
- Avoid organic solvents. Students may determine what the maximum organic solvent concentration can be used with the µPADs if some organic solvents are needed. Most wax printed devices can hold up to around 50-60% methanol and ethanol without leaking.
- Store unused µPADs in a plastic bag to avoid contamination.

*Instructors may decide whether to provide their student teams the following tips, written by students, to help with trouble shooting.*

**Tips for MICROCUREs for students from a student’s perspective:**

- Start by writing down a procedure based on what you understand from the article.
- Try doing the reaction from the article (in solution or on a paper disk).
- If it doesn’t work, look more closely at the reagents used to figure out what the important reactions are.  *What reaction causes the color change?  Was a buffer used to control pH? Are there other reagents needed (redox reaction, etc.)?*
- When doing initial testing on paper, pipette reagents instead of soaking to save time and materials
- Most likely, it's a good idea to scale volumes down from the values in the literature
- Most likely the article will not give exact volumes to use, but rather concentrations. Make sure to calculate how much volume of each reagent will have to be made based on the concentrations required and how much of each you anticipate using.
- Make sure to clearly label all solutions/standards/etc that you make
- Make more volume of reagents than what you will anticipate needing to run the experiment. That is good in case something goes wrong.
- When making reagents, it’s sometimes a good idea to make them more concentrated than the literature values and then dilute them. Sometimes the article concentrations don’t work, so trying the experiment with a higher concentration of reagents may give better results.
- When making a series of standards, make a stock solution and then perform serial dilutions
- When troubleshooting, save some volume of reagents by themselves, that way, if the reaction does not work you don’t have to make all the reagents again.
- Pay attention to see what may be causing the problems, if there are any. For example, maybe the reagents are not completely dissolved, or maybe there is a pH issue that may be negatively interfering with the reaction. Trying the reaction in a more basic or acidic environment may help it occur.

**Micro CURE Check-Off Sheet Master (Instructors)**

This sheet is meant to serve as an instructor’s guide to aid students in developing their research process.

Students may be required to create their own checklists (as a part of their weekly reports) as they progress through the project, getting feedback and guidance from the instructor.

Individual instructors may choose to emphasize certain aspects of the process, and can provide a partial list to students asking them to decide what experiments they would need to design and what data they need to collect to meet the specific goals highlighted.

| **Core Research Area** | **Status** |
| --- | --- |
| Analyte(s)/sample selected and usable |  |
| Colorimetric reagent(s) obtained and functional (solution tested) |  |
| Reaction mechanism + other reagents known |  |
| PAD layout/design known |  |
| Protocol developed |  |
| Device(s) calibrated |  |
| Standards prepared |  |
| Standard curve created |  |
| FIGURES OF MERIT: |  |
| - Precision |  |
| - Accuracy |  |
| - Sensitivity |  |
| - Linearity |  |
| - Linear range |  |
| - Limit of detection |  |
| - Selectivity |  |
| Repeat samples |  |
| Controls included |  |
| Time testing |  |
| Storage method testing |  |
| Data obtained and organized (graphs, figures, charts, etc.) |  |
| RESEARCH POSTER (cross out sections if they are not to be included in your poster): |  |
| - Title |  |
| - Abstract |  |
| - Introduction |  |
| - Methods |  |
| - Results |  |
| - Reaction |  |
| - Conclusion |  |
| - Sources of error |  |
| - Future work |  |
| - References |  |
| - Acknowledgements |  |

1. https://www.who.int/bulletin/volumes/95/9/16-187468/en/ [↑](#footnote-ref-1)
